# Applying a system dynamics modelling approach to explore policy options for improving neonatal health in Uganda

**DOI:** 10.1186/s12961-016-0101-8

**Published:** 2016-05-04

**Authors:** Agnes Rwashana Semwanga, Sarah Nakubulwa, Taghreed Adam

**Affiliations:** Information Systems Department, College of Computing and Information Sciences, Makerere University, P.O. Box 7062, Kampala, Uganda; Department of Obstetrics and Gynaecology, College of Health Sciences, Makerere University, Kampala, Uganda; World Health Organization, Health Systems and Innovation, 20 Avenue Appia, 1211 Geneva 27, Switzerland

**Keywords:** Neonatal mortality, Systems thinking, Causal loop diagram, Methods, Child health, Uganda, Dynamic modelling, Policy options

## Abstract

**Background:**

The most recent reports on global trends in neonatal mortality continue to show alarmingly slow progress on improvements in neonatal mortality rates, with sub-Saharan Africa still lagging behind. This emphasised the urgent need to innovatively employ alternative solutions that take into account the intricate complexities of neonatal health and the health systems in which the various strategies operate.

**Methods:**

In our first paper, we empirically explored the causes of the stagnating neonatal mortality in Uganda using a dynamic synthesis methodology (DSM) approach. In this paper, we completed the last three stages of DSM, which involved the development of a quantitative (simulation) model, using STELLA modelling software. We used statistical data to populate the model. Through brainstorming sessions with stakeholders, iterations to test and validate the model were undertaken. The different strategies and policy interventions that could possibly lower neonatal mortality rates were tested using what-if analysis. Sensitivity analysis was used to determine the strategies that could have a great impact on neonatal mortality.

**Results:**

We developed a neonatal health simulation model (NEOSIM) to explore potential interventions that could possibly improve neonatal health within a health system context. The model has four sectors, namely population, demand for services, health of the mothers and choices of clinical care. It tests the effects of various interventions validated by a number of Ugandan health practitioners, including health education campaigns, free delivery kits, motorcycle coupons, kangaroo mother care, improving neonatal resuscitation and labour management skills, and interventions to improve the mothers health, i.e. targeting malaria, anaemia and tetanus. Among the tested interventions, the package with the highest impact on reducing neonatal mortality rates was a combination of the free delivery kits in a setting where delivery services were free and motorcycle coupons to take women to hospital during emergencies.

**Conclusions:**

This study presents a System Dynamics model with a broad and integrated view of the neonatal health system facilitating a deeper understanding of its current state and constraints and how these can be mitigated. A tool with a user friendly interface presents the dynamic nature of the model using ‘what-if’ scenarios, thus enabling health practitioners to discuss the consequences or effects of various decisions. Key findings of the research show that proposed interventions and their impact can be tested through simulation experiments thereby generating policies and interventions with the highest impact for improved healthcare service delivery.

**Electronic supplementary material:**

The online version of this article (doi:10.1186/s12961-016-0101-8) contains supplementary material, which is available to authorized users.

## Background

**Fig. 1:**
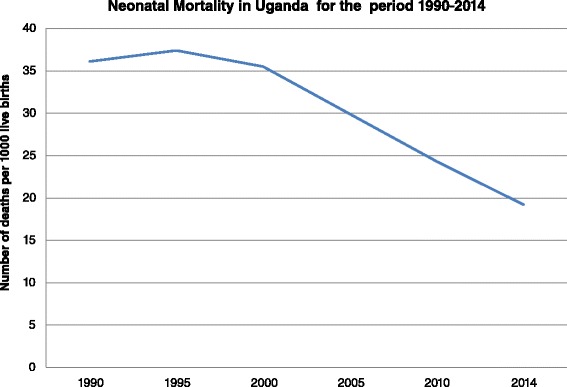
Neonatal mortality rates for Uganda [1]

Alarmingly slow improvements in neonatal mortality rates in most low- and middle-income countries (LMICs) have attracted considerable international attention. According to UNICEF, Africa accounts for 39% of neonatal deaths, with the majority of these deaths occurring specifically in sub-Saharan Africa [1]. Despite the high burden of neonatal mortality, Africa has seen the slowest improvements in neonatal mortality rates with a decline of only 19% from 1990 to 2010 in contrast to the 43% decline witnessed in high-income countries [1]. According to Healthy Newborn Network, more than three quarters of the world’s newborn deaths occurred in sub-Saharan Africa (1,066,000) and South Asia (1,070,000) in 2013 [2]. While some progress in reducing the world’s 3.1 million newborn deaths each year is being made, sub-Saharan Africa is still lagging behind [3]. In Uganda, although the infant mortality rate has reduced considerable from the 156 per 1000 live births in 1995, there is still room for improvement, as shown in Fig. 1.

Although numerous policies in Uganda are specifically targeting the advancement of maternal and neonatal healthcare (see Recent policies and programs that have been used to reduce neonatal mortality in Uganda), there is need for improved local data and analysis, dedicated funding and commitment to achieving high coverage of quality services towards saving lives [3]. For example, the main causes of neonatal deaths, namely severe infections, complications of pre-maturity and tetanus, pre-term birth and neonatal asphyxia, are preventable, but have remained poorly detected, reported and unchanged [4–6]. In addition, many of the underlying causes of neonatal deaths are associated with poor maternal access and utilization of health services. While several case studies have emphasised the complex nature of health systems and implementation of policies [7–9], the experience in Uganda has been that the adoption of evidence-based newborn care practices focus on isolated aspects of neonatal healthcare as opposed to taking a holistic approach that includes all stakeholders involved in the wellbeing of neonates [3].

### Recent policies and programs that have been used to reduce neonatal mortality in Uganda [3]

- Prevention of mother-to-child transmission of HIV – a program integrated through post-natal care service
- Malaria prevention and treatment during pregnancy where mothers are given two doses of Fansidar during the second and third trimesters
- Use of insecticide-treated nets for the prevention of malaria during pregnancy
- Strengthening antenatal care (ANC) through upgrade of health facilities, dissemination of guidelines to units and promoting four ANC visits during pregnancy
- Adolescent and pre-pregnancy care by providing family planning services and minimizing stock outs of family planning drugs
- Care during child birth by encouraging mothers to have skilled attendance during delivery and providing emergency obstetric care to minimize complications during child birth
- Shifting the roles of traditional birth attendants from deliveries to mobilization and support of expectant mothers
- Provision of postnatal care, where mothers return for review 6 months after delivery

Various approaches, including longitudinal studies and mathematical models [10, 11], have been applied to understand problems associated with neonatal mortality, mostly using linear approaches or focusing on parts of the problem [12–16]. However, it has been increasingly recognised that new research that takes into account the intricate complexities of the neonatal health problem is urgently needed [17]. Linear approaches that provide technical solutions are not adequate to mount effective responses, as the adoption and diffusion of innovations which underpin responses to health problems are influenced by complex health systems [18]. This presents the need take into consideration the dynamics, feedbacks and delays associated with the neonatal healthcare system as we attempt to address neonatal healthcare problems.

Systems modelling methodology of Systems Dynamics (SD) is well suited to address dynamic complexity problems in health [19]. SD has been applied in some studies for shared understanding of healthcare problems, hypothesis testing, and generation of scenarios as well as group learning [19–21]. In a study of the complex and dynamic nature of the maternal healthcare system in Uganda, where SD was employed, it was noted that the feedback structures had great policy implications for maternal health with improved maternal mortality rates [8]. In yet another study where SD models were established to predict future need and demand for the paediatric workforce, it was reported that the strength of the SD models were derived from the simulations where policies could be examined and intervened in a timely manner [22].

**Fig. 2:**
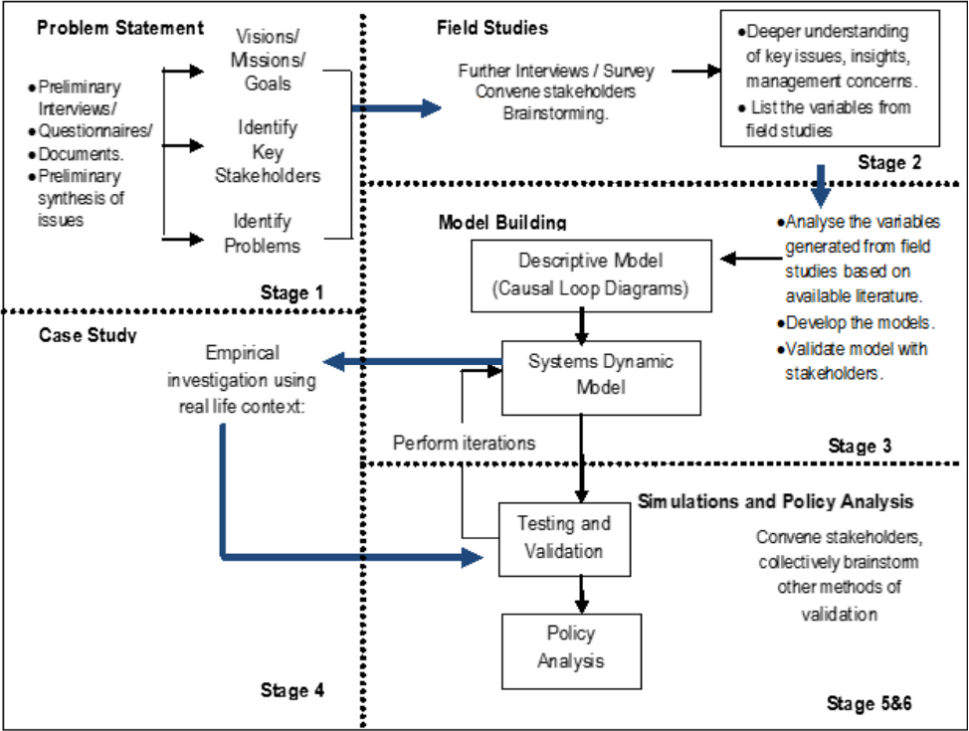
Research design: Dynamic Synthesis Methodology [28, 29]

This research followed the Dynamic Synthesis Methodology (DSM), a SD methodology approach, to holistically study the interrelated and interdependent parts of the neonatal healthcare system as well as the dynamics, feedbacks and delays. The DSM is implemented in six stages, as shown in Fig. 2. In stages 1, 2 and part of 3, we empirically explored the causes of the stagnating neonatal mortality and developed causal loop diagrams that can be used to enhance the understanding of neonatal mortality problems from a feedback view point [7]. This paper presents stages 4, 5, 6 and the last part of 3, which involved the development of a quantitative (simulation) model using STELLA modelling software. The main objective here was to explore how complexity can be examined and taken into account by developing a quantitative (simulation) model for improving policy analysis and design in neonatal health, using STELLA modelling software. The model tests different interventions that could possibly lower neonatal mortality rates as well as determine the strategies that could have a great impact, using Uganda as a case study.

This paper addresses the following questions:

1. What are the requirements for developing a quantitative model that can be used to reveal the unintended consequences of the decisions/actions and mental models (assumptions, views, beliefs, values, etc.) associated with neonatal healthcare?
2. What policy interventions (as well as combinations of these) generated through simulation experiments could possibly lower neonatal mortality rates and by how much?

## Methods

### Study design

SD is a theory of the structure of systems and their resulting dynamic behaviour, whereby structure includes not only the physical aspect of processes but also, importantly, the policies and traditions both tangible and intangible that dominate decision making [23]. The SD methodology was found to be suitable to solve the problems presented by the neonatal health system since it illuminates key principal effects, namely feedback loops, exogenous shocks from sources outside the system they affect (such as changes in demand for neonatal healthcare), systemic delays such as those arising from delivery of health services and unintended consequences resulting from policy changes.

Although many SD modelling approaches have been proposed by other researchers [24–27], the DSM, unlike other approaches, includes a case study which provides empirical investigation and a deeper understanding of the problem. DSM is beneficial in that the strength of case study enables the collection of data in its natural setting and on-site information of the current system from the owners, as well as user requirements and specifications used to develop the model. Figure 2 presents the DSM research design framework by Williams [28] and revised by Rwashana et al. [29], which combines the SD methodology [30–32] and case study methodologies [33] where empirical investigations are done using data obtained from real life contexts (Additional file 1).

DSM has the ability to explore and give insights into complex systems, while being able to use historical data from earlier research to inform the analysis and enhance the development of causal loops and dynamic models. As stated earlier, stages 1, 2 and part of 3 are reported in the first paper [7]. In stage one, preliminary information related to neonatal issues and associated problems was collected from previous studies, historical and statistical reports, and policy documents in order to understand the current problems faced in neonatal healthcare during the first stage. In stage 2, the problem area, objectives and perspectives of the key stakeholders (neonatal experts, mothers, community leaders and healthcare policymakers) were established through interviews, surveys and analysis of data. Interpretation of the issues that were revealed through the interviews and the data collection in stages 1 and 2, together with brainstorming among the authors resulted in the development of two casual loop diagrams (CLDs). These depicted the factors associated with the demand for and supply of health services for neonates and mothers, and also showed the feedback structure of the system, relationships, dynamics, and delays among the various variables and determinants.

In this paper, the implementation of the remaining stages of DSM, namely stages 4–6, is explained.

### Stage 4

**Fig. 3:**
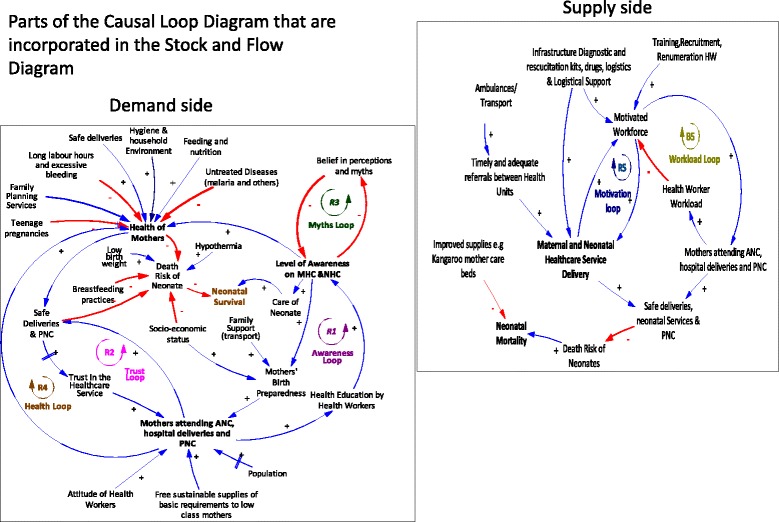
Parts of the causal loop diagram that are incorporated in the stock and flow diagram

A quantitative model (dynamic model) focusing on the dynamic aspects was constructed using STELLA version 8.1 modelling environment (www.iseesystems.com/softwares/Education/StellaSoftware.aspx). The benefits of employing STELLA over other modelling environments include the ability to build dynamic models with basic mathematical and minimal programming skills, the ability to analyse and visualize variables and processes during model building, and the ease of understanding and learning. STELLA software provides a friendly graphical interface and modelling environment for dynamic systems for observing quantitative interaction of variables within a system. The modelling process started off by defining the problems, goals of the model and subsystems based on the state variables and their units as well as the control variables, i.e. those that flow into and out of the state variables and their parameters were selected. The model subsystems of the dynamic model emerged from parts of the two CLDs presented in Fig. 3 [7]. From the CLD showing the demand side, two sectors were generated, one focused on the health of the neonates and mothers while the second was modelled on the factors associated with the demand for healthcare services. The CLD showing the supply side formed the operations sector which models the provision of healthcare services. The population sector presented the dynamics associated with the growth of the population.

The model was examined for possible violations of physical, economic and law of conservation of mass, energy and momentum and the horizon over which the dynamic behaviour of the model was determined. As the model was being run, alternative integration techniques such as reducing the time interval, were employed and examination of the graphs of the variables for reasonableness and data correspondence was performed. Variation of parameters to test the reasonable extremes, whether the results made sense was done while making revisions to address anomalies and errors in the model.

### Stage 5

Empirical investigation with real life data from statistical reports, such as the Uganda Demographic Health Survey reports [34] and national population reports [35], were used to populate the model. The data collected from the reports was inconsistent in some cases where organisations such as the Uganda Bureau of Statistics and the Ministry of Health had different values for the same variable and year. In addition, in some cases, the required data was unavailable and so data for previous years/future years was used and assumptions were made where no data could be found. The data related to health factors from demographic health survey reports was considered more accurate than that from the government, since it is assumed that it is less biased. Once the model had been populated, a comparison of the model results to statistical data was done.

### Stage 6

In the simulations and policy analysis stage, the analysis of the recommendations provided by the respondents in the study guided the development of policy interventions. Three brainstorming sessions where researchers, neonatal and maternal experts convened to discuss a wide range of perspectives, ideas and concerns related to the proposed policy interventions were held [36]. In addition, these sessions were used to test and validate the model. The first session comprised of 12 staff and graduate students from the Bloomberg School of Public Health at Johns Hopkins. The modeller presented the proposed policy interventions and guided the participants through the model with a help of an instrument (Additional file 3) comprised of a description of the model and allowed them to test the model by trying out the modelling exercises. Several ideas and modifications for improved visual display with the aim of making the model more user-friendly and easily understood were proposed as well as key factors that would be useful for demonstrating the dynamics of neonatal healthcare.

After the revisions of the model, we held the second brainstorming session, comprised of six medical staff involved in neonatal and maternal healthcare from three hospitals in Uganda. The different scenarios were tested individually and in combination, resulting in several discussions concerning which intervention would have a great impact. Two new interventions were suggested and these were incorporated in the final model. The third brainstorming session was comprised of eight medical staff, including paediatricians, obstetricians and gynaecologists as well as nursing officers in charge of neonatal and maternity wards. Interventions that could have a great impact on neonatal mortality were further reviewed and the final model was generated.

### System dynamics modelling with STELLA

The model was developed with STELLA Software Research Version 8.1.1. The model focused on the model behaviour such as observing the trend of neonatal mortality rates as well as how low or high the parameter values would go after small changes in the variables during the sensitivity analysis and not on the actual parameter values. The model employed the Euler numerical method, a basic integration method that approximates solutions to differential equations where a small change in variable can result in a significant change in output. STELLA provides four basic elements as explained in Table 1 [32].

**Table 1:** Elements of system dynamics modelling with STELLA

	Description of the element	Symbol
1	***Stocks*** stand for anything that accumulates or drains and these can be physical in nature such as population or non-physical such as trust	
2	***Flows*** are the rate of change of stocks which update the magnitude of stocks and are usually outcomes of decisions by management or external forces [32]	
3	***Converters*** or auxiliary variables are used to take input data and manipulate or convert that input into some output signal; converters may be used for operations (+, –, ÷, ×) and serve as substitutes for either stocks or flows, and include constants, graphical and behavioural relationships	
4	***Connector*** **s** allow information to pass between converters and converters, stocks and converters, stocks and flows, and converters and flows; they serve as inputs and outputs and not inflows and outflows	

STELLA software uses several equations to represent the stock, flow and converters. A brief description of the equations is provided in Additional file 1. The formulation of equations of the model were based on the SD modelling principles [37]:

1. Equations were designed with meaning where variables and parameters correspond to real life meaning
2. Units of equations were tested to ensure that they are dimensionally consistent with the units on the right hand side of the equation corresponding to those on the left hand side
3. Equations were tested to ensure that they yield valid results even in extreme conditions
4. The model was designed to provide realistic description of the real processes where the major focus of the model formulation is the realism and not the mathematical exactness

### Neonatal Healthcare Simulation Model (NEOSIM)

The outcome of these stages was the development of NEOSIM, whose primarily objective at this stage was exploratory to provide a deeper understanding of the dynamic issues that are of concern to managers and researchers as well as provide varying scenarios with different structures and policies. Specifically, the model aimed at illuminating insights and providing understanding of the impact of decisions made on the key variables affecting neonatal healthcare and to aid the decision making process by proposing policies that would enhance maternal and neonatal healthcare services as well as increase health facility deliveries, thereby lowering the neonatal mortality rates. Its anticipated target audience are the stakeholders involved in the neonatal and maternal healthcare operations, management, strategic planning, policy design and implementation as well as research at global, national and district level.

The specific model objectives were based on the following questions:

1. What do we want to achieve?
  - Increased participation in maternal healthcare services (antenatal care (ANC), postnatal care) as well as health facility deliveries
  - Decreased neonatal mortality
  - Improved neonatal and maternal health care services
2. By how much should the level of performance improve? Various scenarios were developed and tested to determine their effect on the selected decision variables

This model focused on the dynamic aspects that may potentially be within control by the stakeholders concerning the demand and provision of neonatal healthcare services. In this model, the duration of a neonate is set to 1 month. The model adopts a simulation time range from 2005 to 2025 years (21 years) since this is a considerably good time for observing the effects of interventions. The input variables (initial values) used in the model are based on the Housing and Population Census reports and Demographic Health Survey reports for Uganda for the last years (Additional file 1). The inputs are presented under the following categories: population growth, demand for maternal and neonatal healthcare, and health service delivery. Population growth variables are associated with the growth of the population while the variables related to the demand are associated with the socioeconomic status and health of the beneficiaries of the health services. The health service delivery factors emerge out of the annual statistical and health reports produced at the national level.

The model makes the calculations based on the input variables resulting in the output variables. The table below presents the key output variables, i.e. the selected key performance indicators that are used to evaluate the level of uptake of maternal and neonatal health services as well as the health service provision (Table 2).

**Table 2:** Key performance indicators

	Key performance indicator	Variable description
1	Uptake of maternal and neonatal healthcare services	Women delivering in health facilities
		Women attending antenatal care
2	Health of the neonates and mothers	Fraction of healthy pregnant women
3	Survival and mortality rates	Neonatal survival rate
		Neonatal mortality rate
		Dying neonates
4	Level of healthcare service provision	Quality of the health system
		Level of access to health facilities
		Level of emergency obstetric care

### The model sectors

The model was divided into the four sectors interacting with each other during the simulation exercise, namely population, health of mothers and neonates, demand, and operations. Some parts of the model, such as the population and healthcare sub-models, are based on common existing models. However, the assumptions, relationships and ideas of the remaining sectors are original ideas of the authors. The detailed explanation of the variables is provided in Additional file 1 and the description and list of equations are provided in Additional file 2.

#### The population sector

The population sector shows the dynamics that are involved in the various population categories. The population sector has a population ageing sub-model with five stocks, which represent the five different age groups, namely neonates (below 1 month), infants (1 month to 1 year), children (1–14 years), reproductive age group (15–49 years) and adults above 50 years. The major outputs of interest emerging from this sector are the number born in a given year (*Neonates*), the number of neonates dying (*DyingNeonates*) and the fraction of the neonates who die over those that are born (*NeonateDyingRate*). According to the simulations, the estimated population for Uganda in 2020 is 47,188,800, which is close to the estimated population of 47,690,000 given by Africapedia without inclusion of the immigrants.

#### The demand sector

The demand sector captures and models the dynamics associated with level of participation in maternal and neonatal healthcare activities which affect the demand. The major outputs for this sector are the level of maternal and neonatal healthcare awareness (*MH&NHCAwareness*), women delivering in health facilities (*HFDeveliveryFraction*) and women attending ANC (*FractWomenAttANC*).

#### The health of mothers and neonates sector

This sector presents the different aspects that contribute greatly to the health of the mothers and neonates. Some of these can be obtained from the health facilities, while others have to be learned from the communities and through health education. The major outputs of the sector are number of healthy pregnant women (*HealthyPregnantWomen*), fraction of healthy pregnant women (*FrHealthPregnantWomen*) and the number of neonates who survive (*NeonatalSurvivalFract*).

#### The operations sector

The operations sector presents the dynamics involved in the supply of maternal and neonatal health services. The input variables captured in this sector include the level of access to health facilities (*HealthFacilityAccessLevel*), the fraction of health workers involved in the government health programmes compared to the required staff level (*HWorkerStaffLevel*), the average health worker skill level compared to the required level (*AvgStafffSkillLevel*) and the level of emergency obstetric care (*LevelEmmergencyObsCare*). The quality of the health system (*QualityOfHealthSystem*), the major output of interest in this sector, affects the trust in the health system, health of the mothers, the number of women having health facility deliveries as well as mothers attending ANC*.*

### Interventions and scenario building

Although a number of interventions related to maternal and neonatal healthcare are found in the literature, a few focused on other aspects of healthcare other than those under the study, for example, a study that looked at adherence to intermittent preventive treatment of malaria among pregnant women [38]. All the interventions found in the literature that focused on the selected key performance indicators were considered in the study. Interventions which could substantially lower neonatal mortality rates were proposed and modelled. Table 3 explains the tested intervention, their sources and estimated impact.

**Table 3:** Tested alternatives

	Intervention described in literature	Results	Reference	Intervention implemented in the model
1	The use of clean delivery kits lowered neonatal infections	Infections of the umbilical cord were reduced by 50%	[39]	Provide motivation package, such as delivery kits, to pregnant women in the communities during delivery with the aim of increasing birth preparedness and skilled birth deliveries; this is in a setting where deliveries in government facilities are free for the population
2	Motorbike ambulances suited to remote areas were provided to the community	Communal referral measures with motorcycle ambulances increased obstetric outcomes, reduced referral time and operating costs in Malawi by 76%	[40]	Motorbike ambulance, where coupons are given to the mothers to ease access to health facilities during delivery
3	Campaigns and strengthening educational interventions	Engagement with regular awareness, educative sessions and collective action to get pregnant women to health units reduced neonatal deaths by 62% in Bolivia	[41]	Use low cost information and communication technologies for increased awareness and to disseminate localized information, such as films during antenatal care sessions, village meetings or worship places; these are regular sessions at least monthly
4	Facility Kangaroo Mother Care (KMC) was used and substantial mortality benefit for babies lower than 2000 g was seen	Effect was 51% reduction in mortality	[42]	Facilitate the implementation of the KMC to prevent neonatal hypothermia; only 10% of health facilities in Uganda had evidence of practicing KMC [3]
5	Regular in service neonatal resuscitation and supervision in China	Regular in service neonatal resuscitation and supervision improved neonatal outcomes and lowered neonatal deaths by 18%	[43]	Provision of regular in service skills in neonatal resuscitation and supervision to ensure this is done correctly and consistently
6	Skills in labour management	Skilled birth attendance reduce neonatal mortality by 25%	[44]	Skills in labour management as well skilled attendance at every delivery; includes monitoring of women in labour using a partograph and intervening correctly and promptly; currently, skilled birth attendance is 68% [33]
7	Malaria prevention	Malaria prevention improves neonatal health by 40%	[45]	Strengthen the use of insecticide-treated nets and provision of intermittent preventive treatment of malaria in pregnancy (IPTp); 52% of the mothers got IPTp during pregnancy [46]
8	Anaemia prevention	Improved neonatal survival by 20–30%	[47]	Increase the coverage of anaemia prevention by giving iron and folic acid to pregnant women; currently, 75% of women receive iron tablets [33]
9	Combination of anaemia and malaria prevention	Reduced the risk of dying of neonates by 76%	[48]	Give both iron/folic supplements and IPTp
10	Tetanus prevention	Tetanus toxoid prevented cases of neonatal death from neonatal tetanus by 43%	[49]	Increase the coverage of tetanus prevention; currently, vaccine coverage for tetanus toxoid is 56% [33]

#### Scenario 1: Incentives for increasing the demand for maternal and neonatal healthcare services (Fig. 4)

Scenario 1 demonstrates the effect of using motorcycle coupons (motorcycle ambulance) to increase the participation to the health facilities, the effect of free clean delivery kits towards increasing birth preparedness and neonatal survival and strengthening of health education campaigns, and the effect of combining the two interventions.

**Fig. 4:**
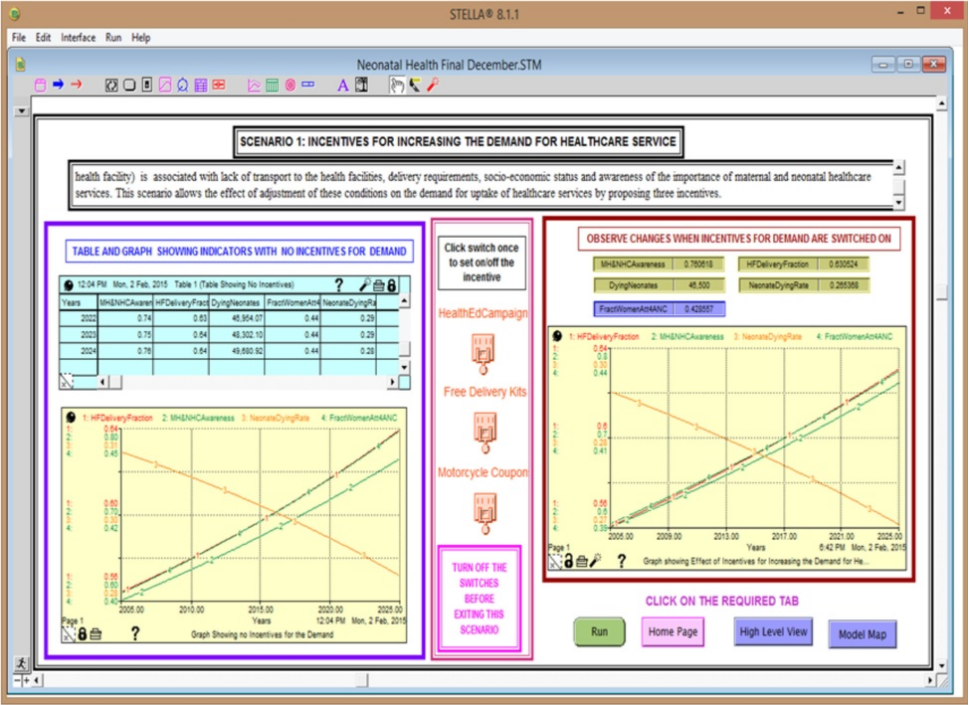
Effects of interventions that can possibly increase the demand for maternal and neonatal healthcare

#### Scenario 2: Interventions targeting the health service delivery

**Fig. 5:**
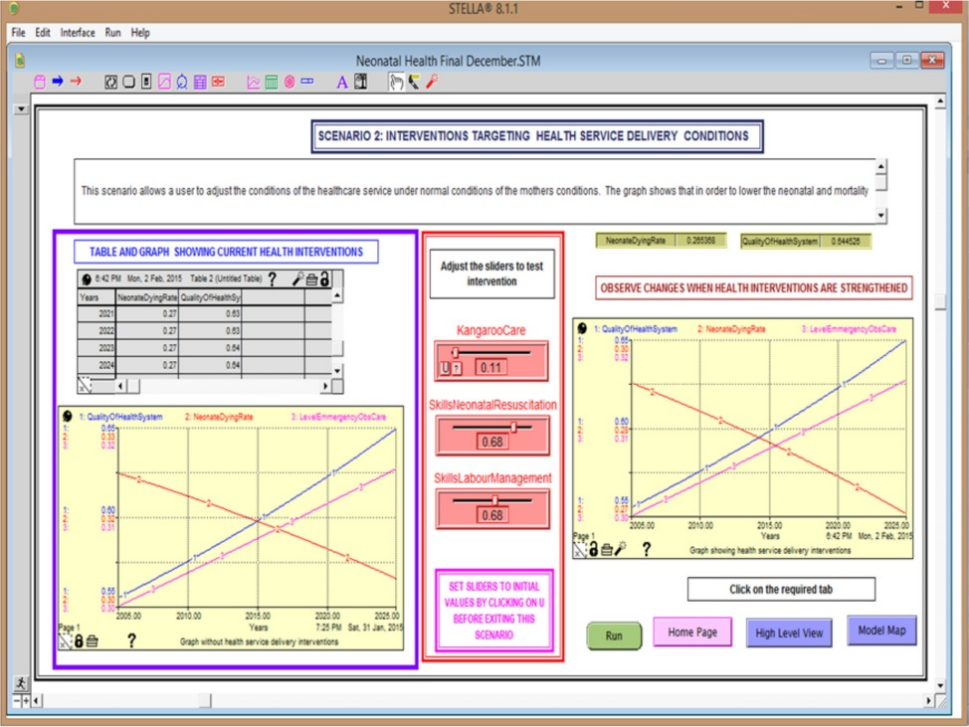
Effect of the interventions targeting health service delivery on neonatal mortality rates

Scenario 2 (Fig. 5) allows the user to adjust the conditions of the healthcare service delivery under the current conditions of the mothers while observing the key performance indicators. In this scenario, we demonstrate the effect of increasing skills in neonatal resuscitation as well as management of labour through refresher training. Labour management can be improved by lowering the skilled midwife to mother ratio in the labour ward, currently at 1:16. We also show the effect of increasing the use of the kangaroo care technique (skin-to-skin contact between infant and parent) towards neonatal death rates.

#### Scenario 3: Interventions targeting the health of pregnant women

**Fig. 6:**
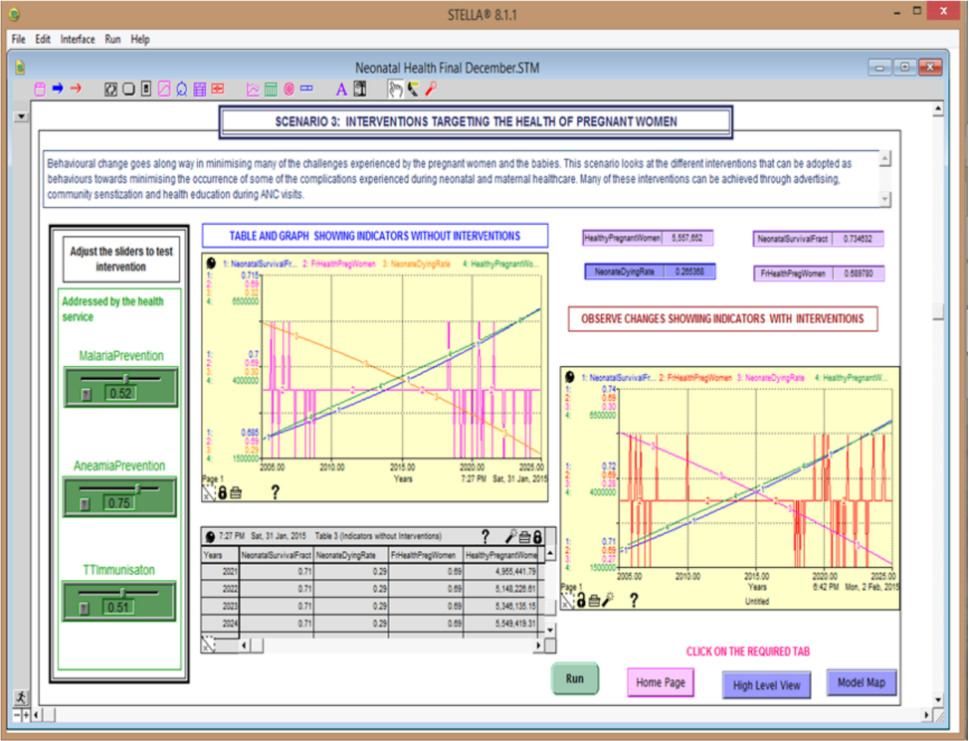
Effect of improving the health of pregnant women on neonatal mortality rates

In scenario 3 (Fig. 6), we demonstrate the effect of malaria and anaemia prevention and tetanus toxoid immunisation as well as several combinations of these on neonatal mortality rates.

### Sensitivity analysis

Sensitivity analysis to identify the interventions with the most significant impact was done to establish key leverage points in the system (the points where small changes to the parameter values cause considerable changes to the behaviour of the values of the outcome-performance measures) thereby providing fundamental and long-term changes to the system. Input variables were selected and tested by multiplying by 0.9 to attain 10% decrease and 1.1 for 10% increase. Since the model has several parameters, the sensitivity was calculated based on the neonatal mortality rates. The sensitivity scale (sensitive (5–14%), very sensitive (15–34%) and highly sensitive (35% and above)) was used to determine the variables that caused a high level of instability.

### Model verification and validation

Model verification and validation were done to ensure that the model has no behavioural errors in relation to the real world and it replicates system behaviour. While verification was performed to ensure that the model is technically correct, validation was performed to make certain that the model structure and assumptions met the purpose for which it was intended. Equations were tested for dimensional consistency, i.e. that units on the right hand side matched those on the left hand side of the equation. All variables and equations of the model were documented and referenced. The model was tested to ensure that the outputs, such as the graphical behaviour and results, were realistic and comparable to historical graphs. Brainstorming sessions with health workers and other researchers to validate the model as well as test the interventions were performed. At the end of the modelling sessions, participants were given an instrument and asked to comment on the experience they had during the modelling session as well as rate the model. Table 4 summarises the participants’ responses to the instruments.

**Table 4:** Results of the validation of the model

Parameter	Rating categories	Responses during the first validation	Responses during second validation	Total responses
How well did they represent issues related to neonatal health services	Very good		2	2
	Good	4	5	9
	Fairly good	2	1	3
	Not at all good			
Whether the model was realistic and an imitation of the real world system	Very reasonable	2	1	3
	Reasonable	4	7	11
	Fairly reasonable			
	Not reasonable			
Does the model capture and communicate issues in neonatal healthcare service?	Very good	3	2	5
	Good	2	5	7
	Fairly good	1	1	2
	Not at all good			
Is the model a useful communication tool concerning neonatal health care issues?	Very useful	4	4	8
	Useful	2	4	6
	Fairly useful			
	Not at all useful			
Is the model a tool that can be used by stakeholders in decision making?	Very useful	3	5	8
	Useful	1	3	4
	Fairly useful	2		2
	Not at all useful			

Participants tried different what-if scenarios by changing the sliders and suggestions on the improvement of the model. The results in the table show that the model was found to reliably capture and communicate issues related to healthcare, was realistic and provided a good imitation of the health system as well as a useful communication tool generating vibrant discussions during the workshop. In addition, the model appropriately represented neonatal health issues and participants agreed that the model could be used by stakeholders for decision making. When asked to comment on the strengths and weaknesses of the model. The majority of participants stated that the model provided a wealth of very important information that addresses issues that could improve the neonatal healthcare services. Two of the participants stated that the model was customized and easy to use by planners and decision makers. One participant, however, stated that the model was sophisticated and therefore needed to be further simplified. Another concern was that the model was dependent on primary statistics that may not reflect real clinical data.

## Results

**Fig. 7:**
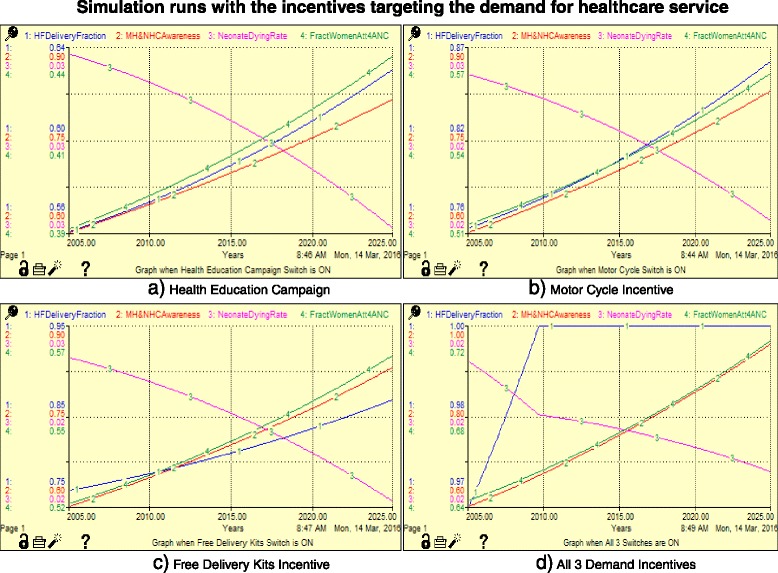
Graphs showing the simulation runs with the incentives targeting the demand for healthcare service

The model results are discussed in relation to three scenarios, as shown in Table 3. In the first instance, the model focuses on the dynamics associated with the demand for healthcare services. Next, it focuses on the interventions targeting health service delivery. Finally, it examines interventions targeting the health of pregnant women. Figure 7 presents the simulation runs for the incentives targeting the demand for health services. All the graphs demonstrated increased health facility deliveries, awareness and fraction of women attending ANC with variations depending on the incentive. Table 5 presents the simulation runs for interventions associated with improved uptake of neonatal healthcare services. It shows that increasing community health education alone does not have a significant impact on the neonatal death rate; however, integrating this with free delivery kits or motorcycle coupons does. A combination of all the three demand incentives results in 100% health facility deliveries after the first 5 years, when introduced with a steady increase in the awareness and fraction of women attending ANC, as shown in Fig. 7. The uptake of maternal health services requires funds for transportation and supplies. While pregnant women may have the knowledge about the importance of health services, they may not be able to deliver in health facilities because of a lack of provision due to the prevailing socioeconomic status.

**Table 5:** Simulation runs for variables associated with the demand interventions

Inputs/Outputs	Switch	Maternal and neonatal healthcare awareness	Health delivery fraction	Neonatal dying rate	Fraction attending four antenatal care visits	Percentage decrease in neonatal dying rate)
Values with no incentives (Base case)		0.7583	0.6184	0.0272	0.4231	
Community health Education	ON	0.8150	0.6302	0.0271	0.4325	0.36%
Motorcycle coupons	ON	0.8281	0.8614	0.0232	0.5617	14.71%
Free delivery kits	ON	0.8300	0.8680	0.0231	0.5650	15.07%
Community health Education + free delivery	ON	0.8929	0.8861	0.0228	0.5755	16.23%
Community health education + Motorcycle	ON	0.8908	0.8794	0.0229	0.5718	15.85%
Motorcycle + free delivery	ON	0.8934	1.0	0.0209	0.7022	23.21%

**Fig. 8:**
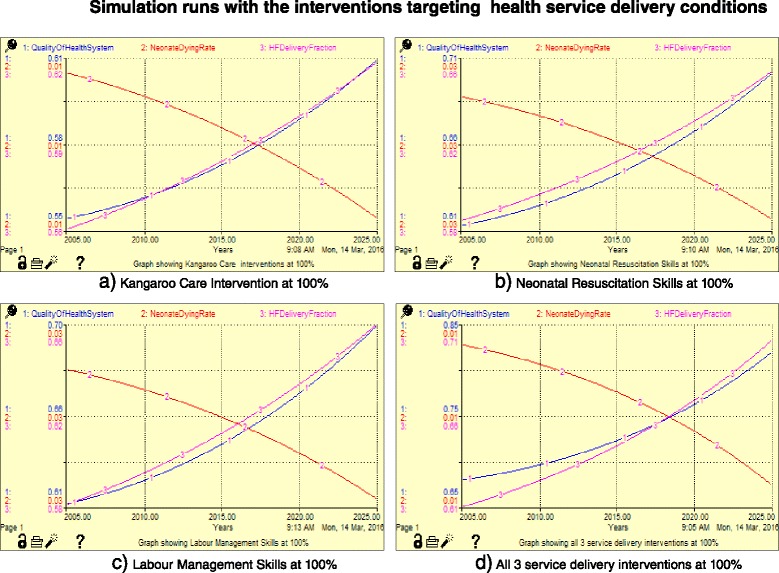
Graphs showing the simulation runs with the interventions targeting health service delivery conditions

Table 6 shows the results of the simulation experiments targeting health service delivery. The interventions presented in this scenario are kangaroo mother care for preterm babies, skills in neonatal resuscitation and labour management. The interventions were modelled based on the results from other studies presented in Table 3. Figure 8 presents the simulation runs for the interventions directed towards improvement in the provision of health services. All the graphs demonstrate a steady increase in the health facility deliveries and quality of the health system and a steady decrease in the neonatal dying rates, with variations depending on the incentive. A combination of all the three interventions (Fig. 8d) shows graphs with steeper curves for all the three variables compared to the remaining graphs (Fig. 8a–c). This further demonstrates the quicker progress in lowering the neonatal dying rates that is brought by combining the three interventions as shown in Fig. 7. The interventions in Table 6 demonstrated low sensitivity (below 5%), which implies that even if these interventions were applied, they would not yield considerable reduction in neonatal death rates. This intervention will be successful if the mothers have transport facilitation and are able to pay the health facility fees. This demonstrates that improving healthcare services without improving uptake is unlikely to lower neonatal deaths.

**Table 6:** Sensitivity experiments for variables targeting health service delivery

Outputs	Base case values		Quality of health service	Health delivery fraction	Neonatal dying rate	Sensitivity scale (based on neonatal dying rate)	Level of sensitivity
Values with no interventions			0.6139	0.6183	0.0272		
Kangaroo care	0.10	–10%	0.6139	0.6183	0.0274	0.73%	Low sensitivity
		+10%	0.6139	0.6183	0.0271	0.36%	Low sensitivity
Skills in neonatal resuscitation	0.68	–10%	0.5964	0.6112	0.0277	1.84%	Low sensitivity
		+10%	0.6112	0.6112	0.0276	1.47%	Low sensitivity
Skills in labour management	0.68	–10%	0.5956	0.6109	0.0276	1.47%	Low sensitivity
		+10%	0.6324	0.6258	0.0269	1.10%	Low sensitivity

The results in Table 7 show the simulations of interventions targeting the health of the mothers. Although the simulation results showed reduced neonatal death rates for increased malaria and anaemia prevention and tetanus toxoid immunisations, the impact shown by the level of sensitivity was rather low. Simulations with 100% compliance of malaria prevention, tetanus toxoid immunisation and anaemia prevention alone resulted in neonatal death rates of 18.5/1000, 28.2/1000 and 23.4/1000, respectively. Simulations with 100% coverage for the three interventions combined resulted in a neonatal death rate of 13.9/1000. The provision of malaria prevention alone would minimise the incidences of malaria sickness, but this would not prevent the mothers from having tetanus, which contributes greatly to the infections. Similarly, some of the mothers are not able to eat nutritious food due to poor economic levels and therefore anaemia prevention still remains essential. These results demonstrate the importance of having compliance of all the three interventions.

**Table 7:** Sensitivity experiments targeting the health of the mothers

Outputs	Base case values		Fraction of pregnant women	Neonatal dying rate	Sensitivity scale (based on neonatal dying rate)	Level of sensitivity
Values with no intervention			0.6898	0.0272		
Malaria prevention	0.52	–10%	0.6773	0.0282	3.68%	Low sensitivity
		10%	0.7023	0.0263	3.31%	Low sensitivity
Tetanus toxoid immunisation	0.51	–10%	0.6825	0.0273	0.37%	Low sensitivity
		10%	0.6971	0.0272	0.0%	Low sensitivity
Anaemia prevention	0.75	–10%	0.7161	0.0289	6.25%	Low Sensitivity
		10%	0.6992	0.0261	4.04%	Low sensitivity
Full compliance with:						
100% malaria prevention		1.0	0.8150	0.0185	–	
100% tetanus toxoid immunisation		1.0	0.7599	0.0282	–	
100% anaemia prevention		1.0	0.7210	0.0234	–	
100% for all the three interventions			0.9063	0.0139	–	

A comparison of the results from Tables 5–7 shows that the interventions directed towards improving the socioeconomic status (transport, requirements for birth preparation) have a greater impact on improving neonatal mortality rates compared to interventions targeting service delivery. This implies that the current problem with neonatal mortality requires a focus on how to reach more women by making health facilities accessible, rather than only focusing on improving healthcare delivery and clinical practice. Simulation results from the last rows of Tables 5 and 7 show that a combination of two or more interventions produces a greater impact towards improving neonatal healthcare.

## Discussion

This study presents the first of its kind SD model that aims to understand the complex dynamics associated with neonatal healthcare. More specifically, this study sought to explore the requirements for developing a quantitative model that can be used to reveal the mental models associated with neonatal healthcare and the complex and unintended consequences of a combination of current policies and interventions. Through a stock and flow model, policy options with the potential of improving maternal and neonatal health were built in and tested in various scenarios and combinations by a group of Ugandan health practitioners and decision makers.

The study led to interesting insights and recommendations to improve neonatal healthcare and to increase the uptake of health services in Uganda. For example, the analysis demonstrated that socioeconomic interventions that are geared towards increasing the uptake of health services are the most interesting of all the interventions tested since they have tremendous impact on increasing health facility deliveries, thereby lowering neonatal deaths. The model also demonstrated the benefits of using motorcycle ambulances that are owned by health facilities to transport the mothers who cannot afford the costs of transportation to the health facilities. In addition, it confirms that health facilities that are well equipped with skilled personnel, drugs and supplies have the potential to build the community’s trust in healthcare services and increase their use. This is also true for having good access to well-equipped health facilities. Finally, interventions which focused on improving availability of neonatal resuscitation skills, labour monitoring skills and kangaroo care also demonstrated reduction in mortality rates to substantially low levels.

One of the strengths of our model is that it provides a broad and integrated view of the neonatal health system, which facilitates a deeper understanding of its current state and constraints and how they can be mitigated. It provides a tool with a user friendly interface, despite the underlying complexity of the analysis and model characteristics. The dynamic nature of the model using ‘what-if’ scenarios in various combinations helped the decision makers and health practitioners who tested the model to immediately see and discuss the consequences or effects of various decisions. These multiple simulation experiments facilitated policy modelling and improvement by the participants, which created engagement and enriched the discussion and recommendations of best policy options, consistent with the findings by Maani and Cavana [32]. The model also helped illuminate key processes and aspects in the neonatal health system, which was found useful for process improvement and operational management.

There are several limitations to this model, however. The first is that it did not include a costing or cost effectiveness component for the various tested scenarios. The range of interventions included in this first exploratory phase is also limited. In addition, we deliberately did not consider interventions that are known to be successfully implemented in Uganda such as the programme to eliminate mother-to-child transmission of HIV. The assumption is that the gains in the operation of service delivery will be maintained. Future work should include other interventions, including those already in place, to more realistically test and estimate the overall impact of packages of interventions on improving neonatal health outcomes. Furthermore, it would be valuable to explore ways to include a qualitative component to the model, acknowledging that quantitative data alone are not sufficient to account for the complexity inherent in the health conditions involved and the health systems within which policy options are considered.

## Conclusion

This paper employs the SD method as an alternative solution towards providing a broad integrated view of the neonatal health system showing the intricate complexities as well as providing a tool that can be used to test interventions and their impact on key variables. NEOSIM, a SD model, has a user friendly interface that enables the testing of ‘what-if’ scenarios, thus enabling health practitioners to discuss the consequences or effects of various decisions. Proposed interventions and their impact can be tested through simulation experiments, thereby generating policies and interventions with the highest impact for improved healthcare service delivery. NEOSIM was found to be a useful tool for communication and was appreciated by the various stakeholders involved in the brainstorming and validation stages, particularly health practitioners and decision makers.

Further piloting of the model with a larger group of stakeholders and a bigger set of policy options would be worth considering for future research in this area. Combinations that include interventions known to be effective and widely implemented, a cost element, as well as exploring the addition of a qualitative component to provide a more comprehensive assessment would result in increased desirability, efficiency and expected impact of the various policy options.

## Additional files

Additional file 1: Description of variables. (DOCX 31 kb) Additional file 2: Description of equations and list of model equations. (DOCX 34 kb) Additional file 3: Model verification and validation instrument. (DOCX 1088 kb)
