# Supplementary material for: Applying a system dynamics modelling approach to explore policy options for improving neonatal health in Uganda
Source: Health Res Policy Syst. 2016 May 4;14:35. doi: 10.1186/s12961-016-0101-8 (PMC4855338; doi:10.1186/s12961-016-0101-8)
Supplement: Additional file 1: — Description of variables. (DOCX 31 kb) [file 12961_2016_101_MOESM1_ESM.docx]

**Appendix – Description of Variables**

Table 1 : Population variables

|  | **Variable Name** | **Description** | **Initial Value** | **Units** | **Reference** |
| --- | --- | --- | --- | --- | --- |
| 1 | AdultsAbove50 | Number of adults above 50 years. The number of adults above 50 years was estimated at 7,410,000 | 7,410,000 | People | UBOS,2002 |
| 2 | Children | Number of children below 15 years up to 1. The number of children aged between 15 and 1 year. | 10,621,000 | People | UBOS,2002 |
| 3 | Infants | Number of infants over 1 month and upto 1. The number of infants below 1 year were estimated at 988,000. (988,000-neonates(82,333)=905,666) | 905,666 | People | UBOS,2002 |
| 4 | Neonates | Number of neonates (infants who are less than a month old or 28 days old). The number of neonates for the year were estimated at 82,333 in 2002. The model assumes that there were 82,33 neonates in 2000. | 82,333 | People | UBOS,2002 |
| 5 | ReproductiveAgeAdults | This is reproductive population aged between 15-49 years. According to UBOS 2002 the reproductive population is estimated at 5,681,000. | 5,681,000 | People | UBOS,2002 |
| 6 | AnnualBirthRate | This is the birth rate per 1000 people. The following birth rates were used in the model | 2000(48.04); 2001 (47.52), 2002 (47.15), 2003(46.57), 2004 (46.31), 2005 (47.39), 2006 (47.35), 2007 (48.12), 2008 (48.15), 2009(47.84), 2010 (47.55), 2011 (47.49), 2012(47.38). | 1/year | http://www.indexmundi.com/g/g.aspx?c=ug&v=25 |
| 7 | ChildMortRate | The rate at which children die per year | 0.0128 | 1/year | UBOS (2002) |
| 8 | DurationAdultAbove50 | The duration it takes for an adult to stop being productive. | 35 | year | -- |
| 9 | DurationChildren | The duration it takes for an infant to be able to become a child. | 11/12 | year | -- |
| 10 | DurationNeonates | Time it takes a neonate to become an infant. | 1/12 | year | -- |
| 11 | DurationReprod | Duration it takes for a child to become reproductive. | 14 | year | -- |
| 12 | FractionOfReprodFemales | The fraction of the females who are reproductive | 0.51 |  | Uganda National Household survey Report 2009/2010 |
| 13 | ReprodAgeMortality | The rate at which people from reproductive age group die per year | 0.0128 | 1/year | UBOS (2002) |
| 14 | InfantMortalityRate | The rate at which infants die per year. According to 2002 Uganda Population Census Report UBOS(2005), the Infant Mortality Rate (IMR) was 122 per 1000 (0.0122) in 1991 and has declined to 0.0097(1995), down to 0.0083(2002). | GRAPH(TIME) | 1/year | UBOS (2002) |

Table 2 : Factors associated with the demand for maternal and neonatal healthcare

|  | **Variable Name** | **Description** | **Initial Value** | **Units** | **Reference** |
| --- | --- | --- | --- | --- | --- |
| 1 | MH&NHCAwareness | Being informed or having knowledge about antenatal care and hospital deliveries. Awareness represents the knowledge of birth preparedness which improves through sensitization, campaigns, literacy levels and is reduced by belief in myths. The model assumes 60% was aware of the importance of antenatal care and health facility deliveries. | 0.6 | Unitless | Field study |
| 2 | WomenAttendingANC | This represents the number of women who attend receive antenatal care at the hospital. 92% of the Ugandan women receive ANC from a skilled provider (684,700 x 92%) | 547,760 | people | UDHS (2011) |
| 3 | DistanceToFacility | Long distances to the health facilities prevent mothers from having health facility deliveries. According to UBOS (2006), 59% births are not from health facilities. 40% of mothers who donot have health facility deliveries stated it was due to the long distance to health facilities. | 0.59 | unitless | UBOS (2006) |
| 4 | WomenDeliveringHF | This is the population of women who have hospital deliveries. According to the AHSPR 2010-11, of the births, 33% were hospital deliveries in 2009, while 2010 had 39%. Based on that statistic, the model assumes that 39%.of the women had hospital deliveries in (684,700 x 39%)=273,600. | 273,600 | people | AHSPR (2011) |
| 5 | BeliefMyths | Ratio of women population that believes in myths. According to the study 14% of the women knew peers in the community who did not have hospital deliveries because of their belief in myths. The model assumes that 1% of the women population believe in myths. | 0.01 | unitless | Field study |
| 6 | EffectForgetingAwareness | The effect of forgeting antenatal care schedules and neglecting health facility deliveries | 0.1 | 1/yr | Assumed |
| 7 | HFAttendanceDemandFraction | This is the fraction of mothers who are well prepared for birth and have their deliveries in health facilities. |  | Unitless |  |
| 8 | MakeItOnsetLabourNight | Onset labours which happen during the night prevent mothers from having health facility deliveries. According to UBOS (2006), 59% births are not from health facilities. 15% of mothers who donot have health facility deliveries stated it was due to onset labours which happen during the night [UBOS, 2006] | 0.15 | Unitless | UBOS (2006) |
| 9 | Mothers'BirthPreparedness | Level of participation in health facility deliveries and antenatal care is dependent on awareness through sensitisation, health education talks, socio-economic status, and access to health facilities. 47% of the women attend at least 4 ANC. | 0.47 | Unitless | UBOS (2006) |
| 10 | Motorcycle_Coupons | Currently 18% pregnant women cannot afford transport to health facilities for deliveries. If motor cycle coupons are provided, this would enable majority of the pregnant women be able to attend health facility deliveries. | 0.1 | Unitless | Field study |
| 11 | MotorcycleTransport | Lack of transport prevents mothers from having health facility deliveries. According to the study, 59% births are not from health facilities and 18% of mothers who do not have health facility deliveries stated it was due to unavailability of transport. According to AHSPR(2010/11), 57% of population do not have access to health facilities. The model assumes (59% x 18% = 0.106) | 0.11 | Unitless | UBOS, 2006  AHSPR (2011) |
| 12 | TrustInHealthService | Lack of trust in the health service (poor attitudes of health workers, lack of basic equipment and drugs) prevent mothers from having health facility deliveries. 59% births are not from health facilities. 18% of mothers who do not have health facility deliveries stated it was due to other reasons including lack of trust in the health service implying that 82% have trust in the health service. | 0.82 | Unitless | UBOS, 2006 |
| 13 | Table_for_average_Ugandan_literacy | This is the table for Ugandan literacy rates. According to Uganda population census 2002 report, 54% of Ugandans were literate in 1991 and 68% were literate in 2002. |  | GRAPH(TIME unitless | UBOS (2005), |

Table 3 : Factors associated with health of the mothers

|  | **Variable Name** | **Description** | **Initial Value** | **Units** | **Reference** |
| --- | --- | --- | --- | --- | --- |
| 1 | AneamiaPrevention | This refers to the pregnant mothers who receive the iron tablets for prevention against anaemia. 63% of the pregnant mothers received iron tablets. According to AHSPR 2010/11, 46% of women have anaemia related diseases. | 0.63 | Unitless | UBOS (2006),  AHSPR (2011) |
| 2 | BirthWeight | Low birth weight babies as a fraction of births is 12% which implies that 88% of the births have the required average birth weight. The average percentage of birth weight of the neonate in Uganda in 2000 was | 0.6 | Unitless | http://www.tradingeconomics.com/uganda/low-birthweight-babies-percent-of-births-wb-data.html. |
| 3 | BreastFeedingPractices | Fraction of mothers practicing breastfeeding. According to Mbonye (2012), 80% of the mothers practiced exclusive breast feeding at birth | 0.8 | Unitless | Mbonye (2012) |
| 4 | EffSafeDeliveries | The effect of safe deliveries on the survival rate of the neonate. MEAN(HFDeliveryFraction,QualityOfHealthSystem) | derived | Unitless | calculated |
| 5 | FactorsHealthMothers | This is the fraction of pregnant mothers who are healthy and will not develop obstetric complications. For purposes of modeling it is assumed that healthy mothers will not need Emergency Obstetric Care (EmOC). Only 15% of the pregnant mothers develop obstetric complications and leaving the 85%.The health of the mother is important in determining whether a neonate survives or not. Some of key factors affecting the health of the mothers include poor feeding and nutrition, frequency of deliveries, teenage pregnancies, hygiene and household environment, untreated diseases and mothers having ANC, health facility deliveries and PNC. | derived | Unitless | calculated |
| 6 | HIV&OtherTests | This refers to the pregnant mothers who accept to take the HIV test during pregnancy. According to UBOS (2006),18% of the pregnant mothers accepted the HIV test and yet the antenatal HIV prevalence in Uganda is 6.5%. | 0.18 | Unitless | UBOS (2006) |
| 7 | Hygiene&HouseholdEnvironment | The effect of hygiene and household environment on the health of the mothers. According to AHSPR 2010/11, the key indicator for hygiene is 71% | 0.5 | Unitless | AHSPR (2011) |
| 8 | InsecticideTreatedNets | This refers to the pregnant mothers who sleep under a mosquito insecticide treated net (ITN) as required for prevention of malaria. According to UBOS (2006), 10% of the pregnant mothers used ITNs. | 0.1 | Unitless | UBOS (2006) |
| 9 | MalariaPrevention | This refers to the pregnant mothers who received the recommended doses of IPTp (Fansidar tablets) as recommended for prevention of malaria during the 2nd and 3rd trimester. 17% of the pregnant mothers received the Fansidar tablets. | 0.17 | Unitless | UBOS (2006) |
| 10 | NeonatalSurvivalFract | This is the probability that the neonate will survive and it is determined by minimising the risk factors such as poor breast feeding practices, hypothermia, unsafe deliveries and low birth weight. Mean (BreastFeedingPractices,WarmClothing,BirthWeight,EffSafeDeliveries, FactorsHealthMothers) | derived | Unitless | Calculated |
| 11 | RightReproductiveAge | This refers to the pregnant mothers who become pregnant before the age of 20 presenting a high risk of maternal and neonatal death. According to UBOS (2006), 18.7% of the pregnant mothers were below the recommended age implying that 81.3% of the mothers gave birth at the right time. According to UDHS(2001), the fraction of teenagers (15-19years) having pregnancies was 30%. | 0.813 | Unitless | UBOS (2006)  UDHS(2001) |
| 12 | SufficientDiet&Nutrition | This refers to the pregnant mothers who had the right nutrition/diet during pregrancy. Poor feeding results from poverty and the poverty rate of Uganda in 2000 was 35%. The model assumes that 65% of the pregnant women were having the right diet. | 0.65 | Unitless | UBOS (2006) |
| 13 | TTImmunisaton | This refers to the pregnant mothers who receive the Tetanus immunisation. According to UBOS (2006),51% of the pregnant mothers received tetanus toxoid vaccine. | 0.51 | Unitless | UBOS (2006) |

Table 4 : Factors associated with health service delivery

|  | **Variable Name** | **Description** | **Initial Value** | **Units** | **Reference** |
| --- | --- | --- | --- | --- | --- |
| 1 | AvgStaffSkillLevel | This is the level of staff skill in the various health centres. In 2003, 57% of the health workers were qualified. | 0.57 | Unitless | [Garbus and Marseille (2003)] |
| 2 | HealthFacilityAccessLevel | This refers to the accessibility of health infrastructure to the community to within a radius of 5 kilometers. Health Sector Strategic Plan report (2001) estimates current level of accessibility of the population to health facilities estimated at 49%. There are 5,152 parishes in Uganda. 49% of the parishes have some form of health facility. | 0.49 | Unitless | [Garbus & Marseille(2003)] |
| 3 | HWorkerStaffLevel | This refers to the total number of nurses involved in the government. According to AHSPR 2003/2004, 50% (2627) of the desired positions are filled. (5254). According to AHSPR 2010/11, 55% of the health worker positions were filled. | 0.5 | Unitless | AHSPR (2004)  AHSPR (2011) |
| 4 | LevelEmmergencyObsCare | The level of technology adoption in the health facilities. The model assumes 30% since only the 30% of the health facilities that participated in the study had emergency equipment. | 0.3 | Unitless | Assumed |
| 5 | LevelOfRemuneration | The level of remuneration of health workers. The model assumes that the current level of remuneration in the health facilities is 30% where the desired is 100%. | 0.3 | Unitless | Assumed |
| 6 | LevelSupplies&Drugs | This is the availability of drugs and supplies in health facilities. The model assumes that the current level at which health facilities are fully equipped with supplies and drugs is 40% since 60% of the health facilities in the study reported frequent stock outs. | 0.4 | Unitless | Assumed |
| 7 | AmountTrainingGiven | The model assumes that refresher training for health workers is held for 4 weeks in a year | 4/52 | Year | Assumed |
| 8 | AvailabilityOfAmbulances | The rate at which transport is available in healthcare service per year. | 0.005 | 1/year | Assumed |
| 9 | AvgAttritionRate | This is the outlflow rate of health human capital due to HIV/AIDS, politics, business and other activities for the last 20 years. (30% leave rate for the last twenty years) | 0.3/20 | 1/year | Maniple(2004) |
| 10 | DecayFractConstruction | The rate at which constructed health units depreciate per year. For a 25 year life we calculate (1/25)=0.04 | 0.04 | Unitless | Assumed |
| 11 | DepletionSuppliesRate | This is the rate at which supplies are being depleted. The model assumes that supplies in the health facilities are being depleted at the rate of 3%. | 0.03 | Unitless | Assumed |
| 12 | DesiredHealthFacilityAccessLevel | 100% infrastructure upgrade is required such that each parish has at least some form of health facility ( 100% of the population living within a radius of 5kms of an existing health facility). | 1 | Unitless | Assumed |
| 13 | DesiredSkillLevel | The desired level of health worker skills in the health facilities is 100% | 1 | Unitless | Assumed |
| 14 | FractImprovementInfrastrUpgrade | The rate at which new health facilities are constructed. The growth rate of the health facilities was reported as 6-9% (1996-2000). | 0.06 | Unitless | Min. Health, Health Infrastructure, Development and Maintenance Planning (2001) |
| 15 | HiringRate | Health worker replacements are normally about 20% of the projected target for ten years [Maniple,2004] | 0.02 | {1/year} Unitless | [Maniple,2004] |
| 16 | MaxImpactHourTraining | The impact got from one hour of training a health worker.  The impact got from one hour of training a health worker. The model assumes a value of 10%. | 10/100 | Unitless | Assumed |
| 17 | QualityOfHealthSystem | The effectiveness of the health system refers to the level at which health care system are able to provide services to the community. | derived | Unitless | Calculated |
| 18 | RemunerationRevisionRate | The rate at which the remuneration is revised. The model assumes the that these are revised every 20 years since there has not been a systematic way of handling this. | 0.05 | { 1/yr } Unitless | Assumed |
| 19 | ReplenishingRate | The rate at which supplies are replenished. The model assumes a rate of 5% per year. | 0.05 | Unitless | Assumed |
| 20 | SkillsLabourManagement | Skills in labour management as well skilled attendance at every. The current skill level is 68% | 0.68 | Unitless | Assumed |
| 21 | SkillsNeonatalResuscitation | This is the current level of skills in neonatal resuscitation. The model assumes the current skill level to be 68%. | 0.68 | Unitless | Assumed |
| 22 | StockOutRate | According to AHSPR (2010), the percentage of health facilities with(out) stock outs of the 6 tracer medicines was 43%(57%) | 0.57 | Unitless | AHSPR (2010) |
| 23 | TargetStaffLevels | The required number of nurses. The desired number of nurses is 5254. The model assumes this as the required level to make 80% | 0.8 | Unitless | .[MOH:Annual health Sector Performance Report 2003/4 Oct 04] |
| 24 | TechAdoptionRate | The rate at which technology is becomes obsolete in healthcare service per year. | 0.001 | {1/yr } Unitless |  |
