# Supplementary material for: Applying a system dynamics modelling approach to explore policy options for improving neonatal health in Uganda
Source: Health Res Policy Syst. 2016 May 4;14:35. doi: 10.1186/s12961-016-0101-8 (PMC4855338; doi:10.1186/s12961-016-0101-8)
Supplement: Additional file 2: — Description of equations and list of model equations. (DOCX 34 kb) [file 12961_2016_101_MOESM2_ESM.docx]

**Description of Equations and List of Model Equations**

Description of the equations that are used in the model:

1. *Stock equations* in STELLA are represented by two equations as illustrated below :-

|  | $S\left( t \right)=S\left( 0 \right)+ \int_{0}^{t} \left( bx-dx \right)dt$ simplified to $S\left( t \right)=S\left( t-dt \right)+ \left( I-O \right)dt$ | *(Eq.1)* |
| --- | --- | --- |

Equation (1) represents stock at present time (t) which is equal to the stock at previous time S (t-dt) plus inﬂows (I) less the outﬂows (O) during the period (dt).

1. *Flow Equation* is generally a policy statement in the system reﬂecting the rate at which the system will change during the forthcoming simulation interval of time (i.e. the next DT). For example,

|  | $Births =\frac{\left( Population x BirthFraction \right)}{\mathrm{Time}}$ | *(Eq.2)* |
| --- | --- | --- |

This implies that the number of births of the speciﬁed interval is equal to the population multiplied by the birth rate.

1. *Converter Equation* is an intermediate variable, constant or graphical relationship such as:

linear equations. While linear equations assume that the output is proportional to the input, non-linear equations arise from the dynamic feedback nature of the model where the output is a function of the product of a number of variables. *Product formulations* bring out additive and multiplicative effects with a general form of formulation such as these:

$Y =(\left( EffectOfX_{1}onY \right)+ \left( EffectOfX_{2}onY \right)+ ... + \left( EffectOfX_{n}onY \right)) x Ynormal$ *(Eq.4)*

|  | $Y =\left( EffectOfX_{1}onY \right) x \left( EffectOfX_{2}onY \right) x ... x \left( EffectOfX_{n}onY \right) x Ynormal$ | *(Eq.5)* |
| --- | --- | --- |

where the output Y is an additive or multiplicative function of ($X_{1}$*,*$X_{2}$*,…*$, X_{n}$*)*

In such a multiplicative effect formulation, all functions *f* ( ) must yield 1. Normalization is used to keep the different model parameters within the ranges of the functions since absolute values would make it impossible to build robust experimental models. The model used several of the multiplicative and effect formulation equations to represent the dynamic feedbacks in the model.

**LIST OF MODEL EQUATIONS**

**OPERATIONS SECTOR**

AvgStaffSkillLevel(t) = AvgStaffSkillLevel(t - dt) + (ChangeInSkill_Level) * dt

INIT AvgStaffSkillLevel = 0.57{ unitless}

DOCUMENT: This is the level of staff skill in the various health centres. In 2003, 57% of the health workers were qualified. [Garbus and Marseille (2003)]

INFLOWS:

ChangeInSkill_Level = MEAN(AvgStaffSkillLevel*EffOfTrainingCurrentSkillLevel*EffectSkillLabMgt*SkillsLabourManagement*EffSkillNeonatalResusc*SkillsNeonatalResuscitation)

HealthFacilityAccessLevel(t) = HealthFacilityAccessLevel(t - dt) + (ChangeInInfrastructure) * dt

INIT HealthFacilityAccessLevel = 0.49{ unitless}

DOCUMENT: This refers to the accessibility of health infrastructure to the community to within a radius of 5 kilometers. Health Sector Strategic Plan report (2001) estimates current level of accessibility of the population to health facilities estimated at 49%. There are 5,152 parishes in Uganda. 49% of the parishes have some form of health facility [Garbus & Marseille(2003)].

INFLOWS:

ChangeInInfrastructure = (HealthFacilityAccessLevel*(GapInfrastructureUpgrade*FractImprovementInfrastrUpgrade-DecayFractConstruction)){ fraction/year }

HWorkerStaffLevel(t) = HWorkerStaffLevel(t - dt) + (ChangeHWorkerStaffLevel) * dt

INIT HWorkerStaffLevel = 0.5 {Unitless}

DOCUMENT: This refers to the total number of nurses involved in the government. 50% (2627) of the desired positions are filled. (5254). [MOH:Annual health Sector Performance Report 2003/4 Oct 04]

INFLOWS:

ChangeHWorkerStaffLevel = (HiringRateHW*TargetStaffLevels)-(HWorkerStaffLevel*AvgAttritionRate)

DOCUMENT: The number of people hired or leaving per year, including replacements of health workers leaving as a result of retirement. This is modelled as a biflow - negative quantitiy indicates that staff are leaving while the positive indicates those coming in.

LevelEmmergencyObsCare(t) = LevelEmmergencyObsCare(t - dt) + (ChangeInEmmObstCare) * dt

INIT LevelEmmergencyObsCare = 0.3

DOCUMENT: The level of emmergency obstetric care in the health facilities. The model assumes that the current level of emmergency obstetric care in the health facilities is 30% since only 30% of the health facilities that participated in the study had emergency equipment.

INFLOWS:

ChangeInEmmObstCare = LevelEmmergencyObsCare*(AvailabilityOfAmbulances*EffectOfAmbulanceRate)

DOCUMENT: Rate at which new techniques are being aquired to address emergency obstetric care in health facilities.

LevelOfRemuneration(t) = LevelOfRemuneration(t - dt) + (ChangeInRemuneration) * dt

INIT LevelOfRemuneration = 0.3{unitless}

DOCUMENT: The level of remuneration of health workers. The model assumes that the current level of remuneration in the health facilities is 30% where the desired is 100%.

INFLOWS:

ChangeInRemuneration = LevelOfRemuneration*RemunerationRevisionRate

LevelSupplies&Drugs(t) = LevelSupplies&Drugs(t - dt) + (ChangeInSupplies&Drugs) * dt

INIT LevelSupplies&Drugs = 0.4

DOCUMENT: This is the availability of supplies and drugs. The model assumes that the current level at which health facilities are fully equipped with supplies and drugs is 40% since 60% of the health facilities that participated in the study reported frequent stock outs.

INFLOWS:

ChangeInSupplies&Drugs = LevelSupplies&Drugs*(ReplenishingRate-DepletionSuppliesRate)

AmountTrainingGiven = 6/12 {year}

DOCUMENT: The model assumes that refresher training for health workers is held for 4 weeks in a year

AvailabilityOfAmbulances = 0.05 {1/yr}

DOCUMENT: The rate at which transport is available in healthcare service per year.

AvgAttritionRate = 0.3 { 1/year }

DOCUMENT: This is the outlflow rate of health human capital due to HIV/AIDS, politics, business and other activities for the last 20 years. [Maniple(2004)]. (30% leave rate for the last twenty years)

DecayFractConstruction = 0.04{1/yr}

DOCUMENT: The rate at which constructed health units depreciate per year. For a 25 year life we calculate (1/25)=0.04

DepletionSuppliesRate = 0.03

DOCUMENT: This is the rate at which supplies are being depleted. The model assumes that supplies in the health facilities are being depleted at the rate of 3%

DesiredHealthFacilityAccessLevel = 1 { fraction}

DOCUMENT: 100% infrastructure upgrade is required such that each parish has at least some form of health facility ( 100% of the population living within a radius of 5kms of an existing health facility)

DesiredSkillLevel = 1 { unitless }

DOCUMENT: The desired level of health worker skills in the health facilities

EffectOfAmbulanceRate = 0.05 {1/yr }

DOCUMENT: The effect of having ambulances to emmergency obstetric care. The model assumes an effect of 5% per year.

EffectSkillLabMgt = 1.25

DOCUMENT: Skilled birth attendance reduce neonatal mortality by 25%. Lee et al. (2011)

EffOfTrainingCurrentSkillLevel = MaxImpactHourTraining*AmountTrainingGiven

DOCUMENT: The effect of training on current skill level of health workers.

EffSkillNeonatalResusc = (1+0.18)

DOCUMENT: Skills in neonatal rescuscitation reduced neonatal mortality by 18% (Xui, 2014)

FractImprovementInfrastrUpgrade = 0.0267{ fraction/year}

DOCUMENT: The rate at which new health facilities are constructed. According to Lundberg (2008), the average distance of families to health facitlities improved from 49% (2000) to 73% (2008) i.e (0.73-0.49)/9= 0.24/9=0.0267

GapInfrastructureUpgrade = DesiredHealthFacilityAccessLevel-HealthFacilityAccessLevel{MAX((DesiredHealthFacilityAccessLevel-HealthFacilityInfrastrLevel),0) unitless }

DOCUMENT: The gap between the desired infrastructure upgrade and the current infrastructure upgrade.

Gap_In_Staff_levels = (TargetStaffLevels-HWorkerStaffLevel)

DOCUMENT: The difference between the desired staff level and the current staff level.

HiringRateHW = 0.02{1/year}

DOCUMENT: Health worker replacements are normally about 20% of the projected target[Maniple,2004]

HRPerformance = MEAN(AvgStaffSkillLevel,HWorkerStaffLevel,LevelOfRemuneration)

DOCUMENT: The level of human resource performance is the average effect of the staff skill level, staff level and level of remuneration.

MaxImpactHourTraining = 10/100{ unitless }

DOCUMENT: The impact got from one hour of training a health worker. The model assumes a value of 10%.

QualityOfHealthSystem = MEAN(LevelEmmergencyObsCare,HealthFacilityAccessLevel,HRPerformance,LevelSupplies&Drugs,EffectSkillLabMgt*SkillsLabourManagement,EffSkillNeonatalResusc*SkillsNeonatalResuscitation)

DOCUMENT: The effectiveness of the health system refers to the level at which health care system are able to provide services to the community. This is the average effect resulting from the level of emmergency obstetric care, health facility access level, human resource level of performance, level of supplies and drugs, level of skills in labour management as well as neonatal resuscitation.

RemunerationRevisionRate = 1/20 { 1/yr }

DOCUMENT: The rate at which the remuneration is revised. The model assumes that these are revised every 20 years since there has not been a systematic way of handling this.

ReplenishingRate = 0.05

RequiredCapacity = 10000

SkillsLabourManagement = 0.68

DOCUMENT: Skills in labour management as well skilled attendance at every. The current skill level is 68% (Ref).

SkillsNeonatalResuscitation = 0.68{ Place right hand side of equation here... }

DOCUMENT: This is the current level of skills in neonatal resuscitation. The model assumes the current skill level to be 68% (Ref).

Skill_Shortfall = DesiredSkillLevel-AvgStaffSkillLevel

DOCUMENT: The difference between the staff skill level and the current staff skill level.

TargetStaffLevels = 0.8{ fraction }

DOCUMENT: The required number of nurses.[MOH:Annual health Sector Performance Report 2003/4 Oct 04]. The desired number of nurses is 5254. The model assumes this as the required level to make 80%

**DEMAND SECTOR**

MH&NHCAwareness(t) = MH&NHCAwareness(t - dt) + (ChangeInAwareness) * dt

INIT MH&NHCAwareness = 0.6 {Unitless }

DOCUMENT: Being informed or having knowledge about maternal and neonatal healthcare. Awareness represents the knowledge ofmaternal and neonatal healthcare which improves through campaigns, literacy levels and is reduced by belief in myths. The model assumes 60% was aware of the importance of maternal and neonatal healthcare.

INFLOWS:

ChangeInAwareness = ((EffectCampaignAwareness+(EffHFDelivery*HFDeliveryFraction)+(EffANCAttendance*HFDeliveryFraction)

)-

(EffectForgetingAwareness

*EffectMythsAwareness))*MH&NHCAwareness{ Unitless/time }

WomenAttendingANC(t) = WomenAttendingANC(t - dt) + (ANC_Attendance - HFDeliveries - Other_Deliveries) * dt

INIT WomenAttendingANC = 547760{people}

DOCUMENT: This represents the number of women who attend receive antenatal care at the hospital. According to the UDHS (2011), 92% of the Ugandan women receive ANC from a skilled provider (684,700 x 92%)

INFLOWS:

ANC_Attendance = (ReproductiveAgeAdults*FrOfReprodFemales)*FractWomenAtt4ANC

DOCUMENT: Represents the flow of expectant mothers who attend ANC. These are expectant mothers who visit health facility for antenatal care.

OUTFLOWS:

HFDeliveries = WomenAttendingANC*(HFDeliveryFraction)

DOCUMENT: Expectant mothers who have deliveries in health facilities. . Key factors affecting the attendance of hospital deliveries include (awareness, access to services, socio-economic status) and quality of healthcare system.

Other_Deliveries = WomenAttendingANC*(1-HFDeliveryFraction)

WomenAttendingPNC(t) = WomenAttendingPNC(t - dt) + (PostNatalCareAttendance) * dt

INIT WomenAttendingPNC = 200000{ Place initial value here... }

DOCUMENT: Number of women attending PNC

INFLOWS:

PostNatalCareAttendance = WomenDeliveringHF*FractionAttPNC

WomenDeliveringHF(t) = WomenDeliveringHF(t - dt) + (HFDeliveries - PostNatalCareAttendance) * dt

INIT WomenDeliveringHF = 273600 { people }

DOCUMENT: This is the population of women who have hospital deliveries. According to the AHSPR 2010-11, of the births, 33% were hospital deliveries in 2009, while 2010 had 39%. Based on that statistic, the model assumes that 39%.of the women had hospital deliveries in (684,700 x 39%)=273,600.

INFLOWS:

HFDeliveries = WomenAttendingANC*(HFDeliveryFraction)

DOCUMENT: Expectant mothers who have deliveries in health facilities. . Key factors affecting the attendance of hospital deliveries include (awareness, access to services, socio-economic status) and quality of healthcare system.

OUTFLOWS:

PostNatalCareAttendance = WomenDeliveringHF*FractionAttPNC

AvgForgetingFraction = 0.02{ 1/yr }

DOCUMENT: The fraction of maternal and neonatal healthcare awareness that decays every year.

BeliefMyths = 0.01{ unitless }

DOCUMENT: Ratio of population that believes in myths. The model assumes that 1% of the population believe in myths.

DelayCampaignEffect = 0.25/12 { year }

DistanceToFacility = 0.59

DOCUMENT: Long distances to the health facilities prevent mothers from having health facility deliveries. According to UBOS (2006), 59% births are not from health facilities. 40% of mothers who donot have health facility deliveries stated it was due to the long distance to health facilities [UBOS, 2006]. (0.6)

EffANCAttendance = 0.01

EffectCampaignAwareness = (PULSE(MultiplierEffCampaignAwareness*DelayCampaignEffect,YearOfCampaign))*HealthEdCampaign{unitless}

DOCUMENT: The effect of campaigns on maternal and neonatal healthcare awareness

EffectForgetingAwareness = AvgForgetingFraction*MultiplierEffForgetAwareness

DOCUMENT: The effect of forgeting on immunisation awareness

EffectMythsAwareness = BeliefMyths*MultiplierEffMythsAwareness*EffLiteracyAwareness { unitless }

DOCUMENT: The effect of myths on immunisation awareness

EffFreeDeliveryKits = STEP(0.78,5)

DOCUMENT: 78% of the women reported that they were influenced to adhere to IPTp as a result of the intervention. (Mbonye et al., 2013)

EffHFDelivery = 0.01

EffLiteracyAwareness = MultiplierEffLiteracyAwareness*Table_for_average_Ugandan_literacy {Unitless }

DOCUMENT: The effect of literacy on maternal and neonatal healthcare awareness

EffMotorcycleCoupons = STEP (0.76,5){ Place right hand side of equation here... }

DOCUMENT: The use of motorcyles ambulances improved obstetric outcomes by 76% .

FractionAttPNC = 0.5

FractWomenAtt4ANC = MEAN (DistanceToFacility,QualityOfHealthSystem,MH&NHCAwareness,Mothers'BirthPreparedness,MotorcycleTransport,ProvisionOfDeliveryKits)

Free_Delivery_Kits = 0.7

HealthEdCampaign = 0{ Unitless}

HFDeliveryFraction =

MIN(1,(Mean (DistanceToFacility,TrustInHealthService,QualityOfHealthSystem,Mothers'BirthPreparedness,MH&NHCAwareness))*Mean((1+MotorcycleTransport), (1+ProvisionOfDeliveryKits)))

Mothers'BirthPreparedness = 0.47

DOCUMENT: Level of participation in health facility deliveries and antenatal care is dependent on awareness through sensitisation, health education talks, socio-economic status, and access to health facilities. 47% of the women attend ANC.

MotorcycleTransport = (0.18*0.59)+EffMotorcycleCoupons*Motorcycle_Coupons

DOCUMENT: Lack of transport prevents mothers from having health facility deliveries. According to UBOS (2006), 59% births are not from health facilities. 18% of mothers who donot have health facility deliveries stated it was due to unvailability of transport [UBOS, 2006].

Motorcycle_Coupons = 0.4

DOCUMENT: Currently 18% pregnant women cannot afford transport to health facilities for deliveries. If motor cycle coupons are provided, this would enable majority of the pregnant women be able to attend health facility deliveries.

ProvisionOfDeliveryKits = EffFreeDeliveryKits*(Free_Delivery_Kits) { Unitless}

DOCUMENT: The ratio of the current poverty levels to the required levels where poverty level is the degree of shortage or want in the community. This represents the population that is not able to purchase delivery kits. According to FSPI(2003), 46% of the population lived above the poverty level (56% below)

TrustInHealthService = 0.82*QualityOfHealthSystem

DOCUMENT: Lack of trust in the health service (poor attitudes of health workers, lack of basic equipment and drugs) prevent mothers from having health facility deliveries. According to UBOS (2006), 59% births are not from health facilities. 18% of mothers who donot have health facility deliveries stated it was due to other reasons including lack of trust in the health service (UBOS, 2006]. (0.82)

YearOfCampaign = 5

MultiplierEffCampaignAwareness = GRAPH(TIME{unitless})

(0.00, 0.0115), (1.50, 0.017), (3.00, 0.02), (4.50, 0.024), (6.00, 0.025), (7.50, 0.026), (9.00, 0.0265), (10.5, 0.0265), (12.0, 0.026), (13.5, 0.0185), (15.0, 0.0105)

MultiplierEffForgetAwareness = GRAPH(TIME {Unitless })

(0.00, 0.879), (1.50, 0.843), (3.00, 0.766), (4.50, 0.807), (6.00, 0.748), (7.50, 0.771), (9.00, 0.753), (10.5, 0.69), (12.0, 0.672), (13.5, 0.631), (15.0, 0.627)

MultiplierEffLiteracyAwareness = GRAPH(TIME {unitless })

(0.00, 0.031), (1.50, 0.0335), (3.00, 0.035), (4.50, 0.0405), (6.00, 0.044), (7.50, 0.0505), (9.00, 0.0535), (10.5, 0.056), (12.0, 0.0595), (13.5, 0.061), (15.0, 0.0665)

MultiplierEffMythsAwareness = GRAPH(TIME { Unitless })

(0.00, 0.0315), (1.50, 0.033), (3.00, 0.0395), (4.50, 0.051), (6.00, 0.0615), (7.50, 0.079), (9.00, 0.081), (10.5, 0.0725), (12.0, 0.0585), (13.5, 0.042), (15.0, 0.0365)

Table_for_average_Ugandan_literacy = GRAPH(TIME{unitless})

(0.00, 0.645), (1.07, 0.65), (2.14, 0.65), (3.21, 0.64), (4.29, 0.645), (5.36, 0.655), (6.43, 0.65), (7.50, 0.64), (8.57, 0.645), (9.64, 0.645), (10.7, 0.655), (11.8, 0.67), (12.9, 0.68), (13.9, 0.59), (15.0, 0.67)

DOCUMENT: This is the table for Ugandan literacy rates. According to UBOS (2006), Uganda population census 2002 report, 64% of Ugandans wre literate in 2002 and 68% were literate in 2005.

**HEALTH OF MOTHERS AND NEONATES SECTOR**

AneamiaPrevention = 0.75

DOCUMENT: This refers to the pregnant mothers who receive the iron tablets for prevention against anaemia. According to the situational Analysis 33/39 (84.6%) health facilities offered iron and folic acid to the woem who attended ANC. According to UBOS (2006),63% of the pregnant mothers received iron tablets. Currently 75% of women receive Iron tablets (UDHS, 2011)

BirthWeight = 0.8

DOCUMENT: Low birth weight babies as a fraction of births is 12% which implies that 88% of the births have the required average birth weight. The average percentage of birth weight of the neonate in Uganda in 2000 was http://www.tradingeconomics.com/uganda/low-birthweight-babies-percent-of-births-wb-data.html

BreastFeedingPractices = 0.8

DOCUMENT: Fraction of mothers practicing breastfeeding. According to Mbonye (2012), 80% of the mothers practiced exclusive breast feeding at birth

EffAnaemiaPrevention = (1+0.25)

DOCUMENT: Improved neonatal survival by 20-30% Ramakrishnan et al. (2014). Model assumes 25%.

EffBirthWeight = 1

EffMalariaPrevention = (1+0.4)

DOCUMENT: This refers to the pregnant mothers who received the recommended doses of IPTp (Fansidar tablets) as recommended for prevention of malaria during the 2nd and 3rd trimester. According to UBOS (2006), (17%) of the pregnant mothers received the Fansidar tablets while 52% of the mothers got IPTp during pregnancy (Ndyomugyenyi and Katamanywa, 2010).

EffOfKangarooCare = (1+0.51)

DOCUMENT: KMC reduced mortality by 51%.

EffQualityOfHealthSystem = 1

EffTTImmunisation = (1+0.43)

DOCUMENT: Tetanus Toxoid prevented cases of neonatal death from neonatal tetanus by 43%.

FactorsHealthMothers = MEAN(AneamiaPrevention*EffAnaemiaPrevention,PMTCT,WomenSoughtTreatment,MalariaPrevention,SufficientDiet&Nutrition,RightPregnAge,TTImmunisaton*EffTTImmunisation, Hygiene&HouseholdEnvironment, FPMetNeed, EffMalariaPrevention*MalariaPrevention)

DOCUMENT: This is the fraction of pregnant mothers who are healthy and will not develop obstetric complications. For purposes of modeling it is assumed that healthy mothers will not need Emergency Obstetric Care (EmOC). According to UBOS (2006), only 15% of the pregnant mothers develop obstetric complications and leaving the 85%.The health of the mother is important in determining whether a neonate surivives or not. Some of the factors affecting the health of the mothers include poor feeding and nutrition, frequency of deliveries, teenage pregnancies, hygiene and household environment, untreated diseases and mothers having ANC, health facility deliveries and PNC.

FPMetNeed = 0.6

DOCUMENT: According to the situational analysis the total unmet need for family planning services is 40% implying that the total met need is 60%. Ministry of Health. Situation analysis of newborn health in Uganda: current status and opportunities to improve care and survival. Kampala: Government of Uganda. Save the Children, UNICEF, WHO; 2008.

FrHealthPregWomen = HealthyPregnantWomen/(ReproductiveAgeAdults*FrOfReprodFemales)

HealthyPregnantWomen = ReproductiveAgeAdults*FrOfReprodFemales*FactorsHealthMothers

Hygiene&HouseholdEnvironment = 0.75

DOCUMENT: The effect of hygiene and household environment on the health of the mothers.

KangarooCare = 0.1

DOCUMENT: Only 1 in 10 (10%) of the health facilities in the country had evidence of practising Kangaroo Mother Care (KMC) - (Mbonye et al. 2012). KMC reduced mortality by 51%.

MalariaPrevention = 0.52

DOCUMENT: This refers to the pregnant mothers who received the recommended doses of IPTp (Fansidar tablets) as recommended for prevention of malaria during the 2nd and 3rd trimester. According to UBOS (2006), (17%) of the pregnant mothers received the Fansidar tablets. Malaria prevention improves neonatal health by 40%

NeonatalSurvivalFract = Mean (BreastFeedingPractices,WarmClothing,BirthWeight*EffBirthWeight,QualityOfHealthSystem*EffQualityOfHealthSystem, 1.5*HFDeliveryFraction,FactorsHealthMothers, (KangarooCare*EffOfKangarooCare),(EffMalariaPrevention*MalariaPrevention), (AneamiaPrevention*EffAnaemiaPrevention))

DOCUMENT: This is the probability that the neonate will survive and it is determined by minimising the risk factors such as poor breast feeding practices, hypothermia, unsafe deliveries and low birth weight.

PMTCT = 0.56

DOCUMENT: This refers to the fraction of the health facilities that provide PMTCT (prevention of mother to child transmission of HIV/AIDs from the mother to the babies. According to situational analysis (MOH, 2008), 56% health facilities provided PMTCT to pregnant women.

RightPregnAge = 0.813

DOCUMENT: This refers to the pregnant mothers who become pregnant before the age of 20 presenting a high risk of maternal and neonatal death. According to UBOS (2006), 18.7% of the pregnant mothers were below the recommended age implying that 81.3% of the mothers gave birth at the right time.

SufficientDiet&Nutrition = 0.65

DOCUMENT: This refers to the pregnant mothers who had the right nutrition/diet during pregrancy. According to UBOS (2006), poor feeding results from poverty and the poverty rate of Uganda in 2000 was 35%. The model assumes that 65% of the pregnant women were having the right diet.

TTImmunisaton = 0.51

DOCUMENT: This refers to the pregnant mothers who receive the Tetanus immunisation. According to Situational Analysis as well as UBOS (2006),51% of the pregnant mothers received tetanus toxoid vaccine. Currently vaccine coverage for TT is 56% (UDHS, 2011)

WarmClothing = 0.9

DOCUMENT: The effect of hypothermia on the survival rate of the neonate.

WomenSoughtTreatment = 0.61

DOCUMENT: This refers to the pregnant mothers who seek treatment when they fall sick. According to the situational Analysis (MOH, 2008) 61% of the mothers reported to have sought for treatment when they fell sick.

**POPULATION SECTOR**

AdultsAbove50(t) = AdultsAbove50(t - dt) + (Growabove50 - DyingAbove50) * dt

INIT AdultsAbove50 = 7410000 { Place initial value here... }

DOCUMENT: Number of adults above 50 years. According to UBOS 2002 the number of adults above 50 years were estimated at 7,410,000 (UBOS,2002).

INFLOWS:

Growabove50 = ReproductiveAgeAdults/DurationAdultAbove50

OUTFLOWS:

DyingAbove50 = AdultsAbove50*Above50MortalityRate

Children(t) = Children(t - dt) + (GrowtoChild - GrowtoReproductiveAge - DyingChildren) * dt

INIT Children = 10621000 {people}

DOCUMENT: Number of children below 15 years upto 1. According to UBOS 2002 the number of children aged between 15 and 1 year were estimated at 10,621,000 (UBOS,2002).

INFLOWS:

GrowtoChild = Infants/DurationChildren

OUTFLOWS:

GrowtoReproductiveAge = Children/DurationReprod{people/yr}

DyingChildren = Children*ChildMortRate

Infants(t) = Infants(t - dt) + (GrowtoInfant - DyingInfants - GrowtoChild) * dt

INIT Infants = 905666

DOCUMENT: Number of infants over 1 month and upto 1. According to UBOS, 2002 the number of infants below 1 year were estimated at 988,000 (UBOS, 2002). (988,000-neonates(82,333)=905,666)

INFLOWS:

GrowtoInfant = Neonates/DurationInfant

OUTFLOWS:

DyingInfants = Infants*InfantMortalityRate

GrowtoChild = Infants/DurationChildren

Neonates(t) = Neonates(t - dt) + (NeonatesBeingBorn - GrowtoInfant - DyingNeonates) * dt

INIT Neonates = 62000{people}

DOCUMENT: Number of neonates (infants who are less than a month old or 28 days old). According to UBOS, 2002, the number of neonates were estimated at 82,333. The model assumes that there were 62,000 neonates in 2000.

INFLOWS:

NeonatesBeingBorn = AnnualBirthRate*TotalPopulation

DOCUMENT: The number of births in a year

OUTFLOWS:

GrowtoInfant = Neonates/DurationInfant

DyingNeonates = Neonates*NeonateDyingRate/0.1

ReproductiveAgeAdults(t) = ReproductiveAgeAdults(t - dt) + (GrowtoReproductiveAge - Growabove50 - DyingReprodPeople) * dt

INIT ReproductiveAgeAdults = 5681000 {people}

DOCUMENT: This is reproductive population aged between 15-49 years. According to UBOS 2002 the reproductive population is estimated at 5,681,000 (UBOS, 2002).

INFLOWS:

GrowtoReproductiveAge = Children/DurationReprod{people/yr}

OUTFLOWS:

Growabove50 = ReproductiveAgeAdults/DurationAdultAbove50

DyingReprodPeople = ReproductiveAgeAdults*ReprodAgeMortality

Above50MortalityRate = 0.0128{1/yr}

DOCUMENT: The rate at which people aged 50 and above die per year

ChildMortRate = 0.0128{1/yr}

DOCUMENT: The rate at which children die per year (UBOS, 2002)

DurationAdultAbove50 = 35

DOCUMENT: These are the reproductive years of an adult (from age 15 to 50)

DurationChildren = 11/12{yr}

DOCUMENT: The duration it takes for an infant to become a child.

DurationInfant = 1/12 {yr}

DOCUMENT: Time it takes a neonate to become an infant.

DurationReprod = 14{yr}

DOCUMENT: Duration it takes for a child to become reproductive.

FrOfReprodFemales = 0.51

NeonateDyingRate = (1-NeonatalSurvivalFract)*0.1

ReprodAgeMortality = 0.0128{1/yr}

DOCUMENT: The rate at which people from reproductive age group die per year

TotalPopulation = Neonates+ReproductiveAgeAdults+Infants+Children+AdultsAbove50{ people }

AnnualBirthRate = GRAPH(time{ Place right hand side of equation here... })

(2000, 0.049), (2002, 0.046), (2004, 0.0475), (2006, 0.046), (2008, 0.0455), (2010, 0.0415), (2012, 0.043), (2014, 0.044), (2016, 0.046), (2018, 0.046), (2020, 0.0465)

DOCUMENT: This is the birth rate per 1000 people. The following birth rates were used in the model 2000(48.04); 2001 (47.52), 2002 (47.15), 2003(46.57), 2004 (46.31), 2005 (47.39), 2006 (47.35), 2007 (48.12), 2008 (48.15), 2009(47.84), 2010 (47.55), 2011 (47.49), 2012(47.38). http://www.indexmundi.com/g/g.aspx?c=ug&v=25

InfantMortalityRate = GRAPH(TIME{1/yr})

(2002, 0.00838), (2004, 0.00784), (2006, 0.0079), (2007, 0.00766), (2009, 0.00765), (2011, 0.0073), (2013, 0.007), (2015, 0.00695), (2016, 0.0067), (2018, 0.0067), (2020, 0.0065)

DOCUMENT: The rate at which infants die per year. According to 2002 Uganda Population Census Report, the Infant Mortality Rate (IMR) was 122 per 1000 (0.0122) in 1991 and has declined to 0.0097(1995), down to 0.0083(2002). So this model uses a graph showing a slight decline from 0.0083. (UBOS, 2002)
