# Supplementary material for: Applying a system dynamics modelling approach to explore policy options for improving neonatal health in Uganda
Source: Health Res Policy Syst. 2016 May 4;14:35. doi: 10.1186/s12961-016-0101-8 (PMC4855338; doi:10.1186/s12961-016-0101-8)
Supplement: Additional file 3: — Model verification and validation instrument. (DOCX 1088 kb) [file 12961_2016_101_MOESM3_ESM.docx]

**Overview of the Neonatal Simulation Model (NEOSIM)**

**Introduction**

Neonatal simulation model (NEOSIM) is a tool that simulates the provision and demand for neonatal healthcare service. It presents the dynamic issues that are of concern to managers and researchers through scenarios with different structures and policies highlighting key areas of improvement. The model was built based on the analysed data that was collected through literature, field studies and the causal loop diagrams as reported in the earlier paper by the same authors. The anticipated beneficiaries of the model are the stakeholders involved in the neonatal and maternal healthcare operations, management, strategic planning, policy design and implementation as well as research at global, national and district level. The model’s clear purpose is to aid the decision making process by proposing policies that would enhance maternal and neonatal healthcare services as well as increase health facility deliveries thereby lowering the neonatal mortality rates.

Specifically the model objectives is based on the following questions:

What do we want to achieve?

1. Improved health of pregnant women
2. Increased participation in maternal healthcare services (ANC, PNC) as well as health facility deliveries
3. Decreased neonatal mortality
4. Improved neonatal and maternal health care services

By how much should the level of performance improve? Various scenarios were developed and tested to determine their effect on the selected decision variables.

What constraints must the modeler work within? The constraints that the researcher will work within will be specified.

This model focuses on the dynamic aspects that may be potentially within control by the stakeholders as far as the demand and provision of neonatal healthcare services.

*Model scope and time frame*

A neonate is defined as a newborn baby less than one month (4 weeks) old. It is highly desirable that neonates survive during the first month of life. In this model the duration of a neonate is set to 0-1 month. The model adopts a simulation time range from 2000 to 2020 years.

**Main interface of the model:** Figure 1 shows the main interface of the model (home page). Instructions that guide the user on how to use the model can be accessed on the instructions button. Buttons are used to navigate back and forth to the different model components.

******

Figure 1: Main interface of the model

**High Level View :** Figure 2 shows the sectors of the model and how they relate. The model has four sectors and these are outlined below:

1. *The population sector* shows dynamics associated with the population categories.
2. *The demand sector* captures and models the dynamics that are associated with level of participation in maternal and neonatal healthcare activities which affect the demand.
3. *The health of mothers and neonates sector* presents the different aspects that contribute greatly to the health of the mothers and neonates
4. *The operations sector* presents the dynamics involved in the supply of maternal and neonatal health services.

******

Figure 2: High level view of the model

**Model Map :** This interface presents the model which is built with stocks, flows, converters and connectors.

**Model Verification and Validation Instrument for NEOSIM**

***Preamble****:* This instrument is designed to verify and validate the Neonatal Simulation Model (NEOSIM). Verification ensures that the model is correctly implemented and matches the specification for the given purpose while validation checks the accuracy of the models’ representation of the real system.

***Objectives of the instrument: The instrument will be used to:***

1. Test for clarity: the extent to which the model clearly captures and communicates issues associated with neonatal healthcare.
2. Test whether the model appears to be a reasonable imitation of a real world system.
3. Test whether the model adequately represents the system structure.
4. Compare whether the model input-output transformations to corresponding input-output transformation for the real system

***Target audience***

- Neonatal and maternal health experts
- National health service policy makers

***Guidelines:*** A description of the model and a CD (with the STELLA software and the NEOSIM) model has been provided. A demonstration of how the model works will be provided (30 minutes). Time for questions will be given after which the participants will be able to use the model and respond to the questions in the instrument.

**A) Testing of the Scenarios**

In this section, three scenarios that were incorporated in the model and are tested. Please refer to the NEOSIM model and the model write up as you respond to the questions in the table below:

**Scenario 1: Incentives for increasing the demand for healthcare services**

*Description* : This scenario allows the effect of adjustment of the conditions associated with the demand for uptake of maternal and neonatal healthcare services. Three incentives (health education campaigns, free delivery kits and motorcycle coupons) are proposed in this scenario.


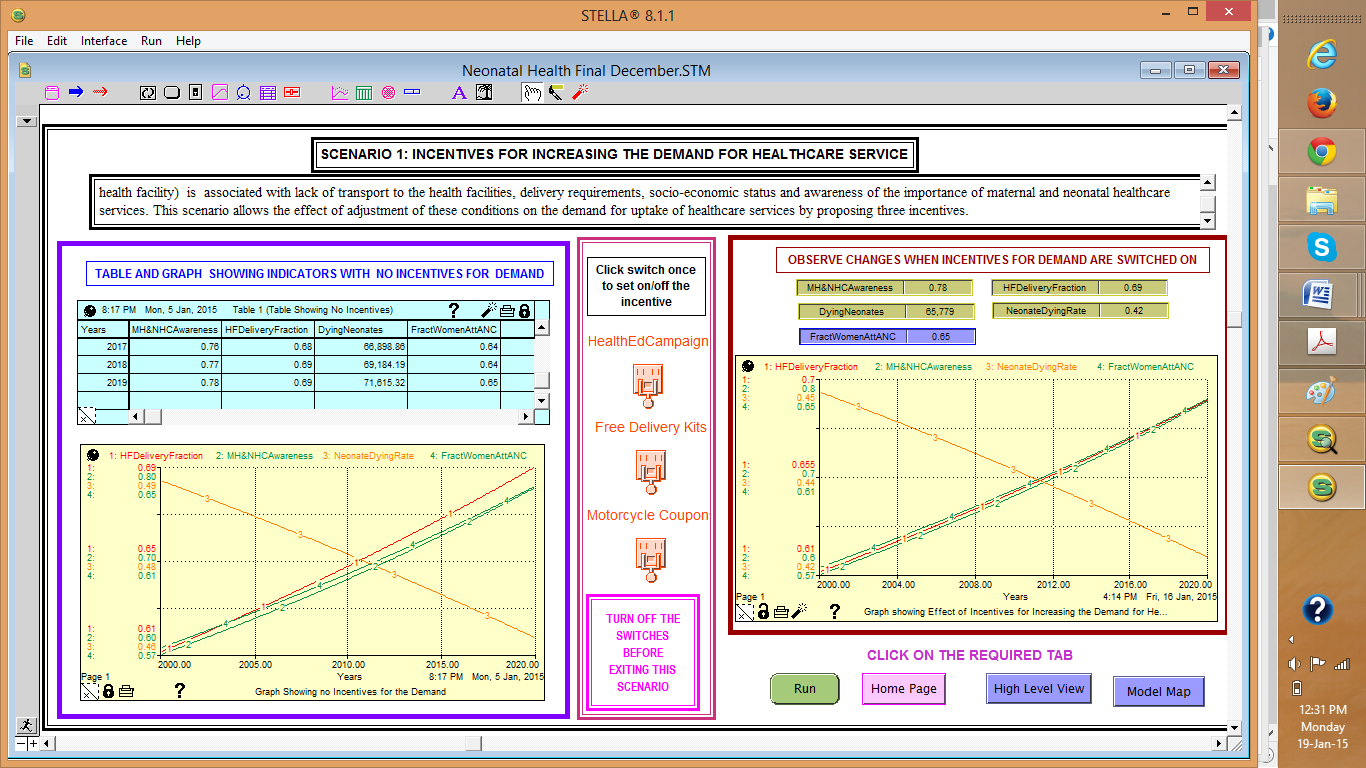


| **Instructions** |
| --- |
| Use switches  to turn the incentives on and off. This will adjust the input variables. Run the model |
| **Provide comments about the behavior and dynamics displayed in the tables and graphs.** |
|  |

**Scenario 2:Interventions targeting health service delivery conditions.**

*Description :* This scenario allows a user to adjust the conditions of the healthcare service. Three interventions (Kangaroo care, skills in neonatal resuscitation and labour management) have been proposed in this scenario.


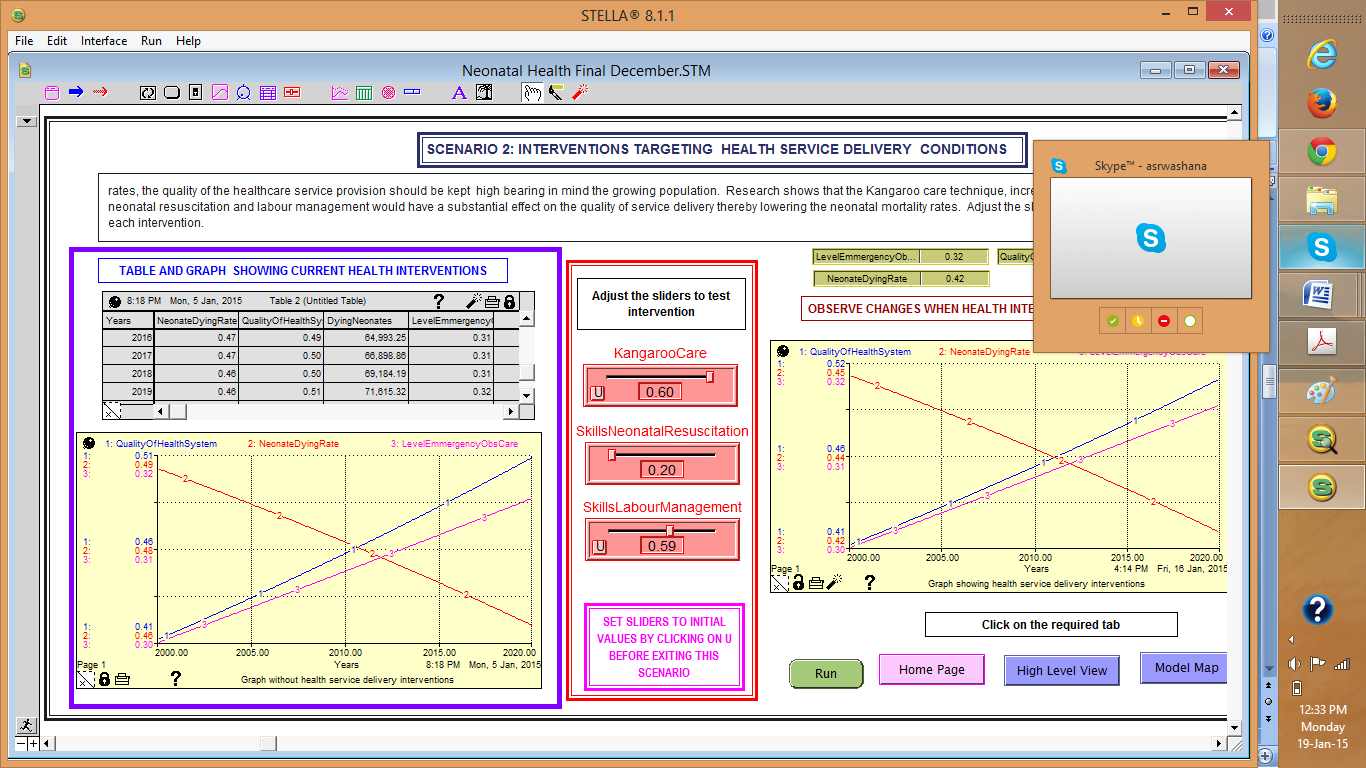


| **Instructions** | |
| --- | --- |
| Use sliders to adjust the input variables, Run the model |  |
| **Provide comments about the behavior and dynamics displayed in the tables and graphs.** | |
|  | |

**Scenario 3:Interventions targeting the health of pregnant women**

*Description :*This scenario looks at the different interventions that can be adopted towards minimising the occurrence of the complications experienced during neonatal and maternal healthcare. Three interventions (malaria prevention, anaemia prevention and Tetanus immunization) have been proposed in this scenario


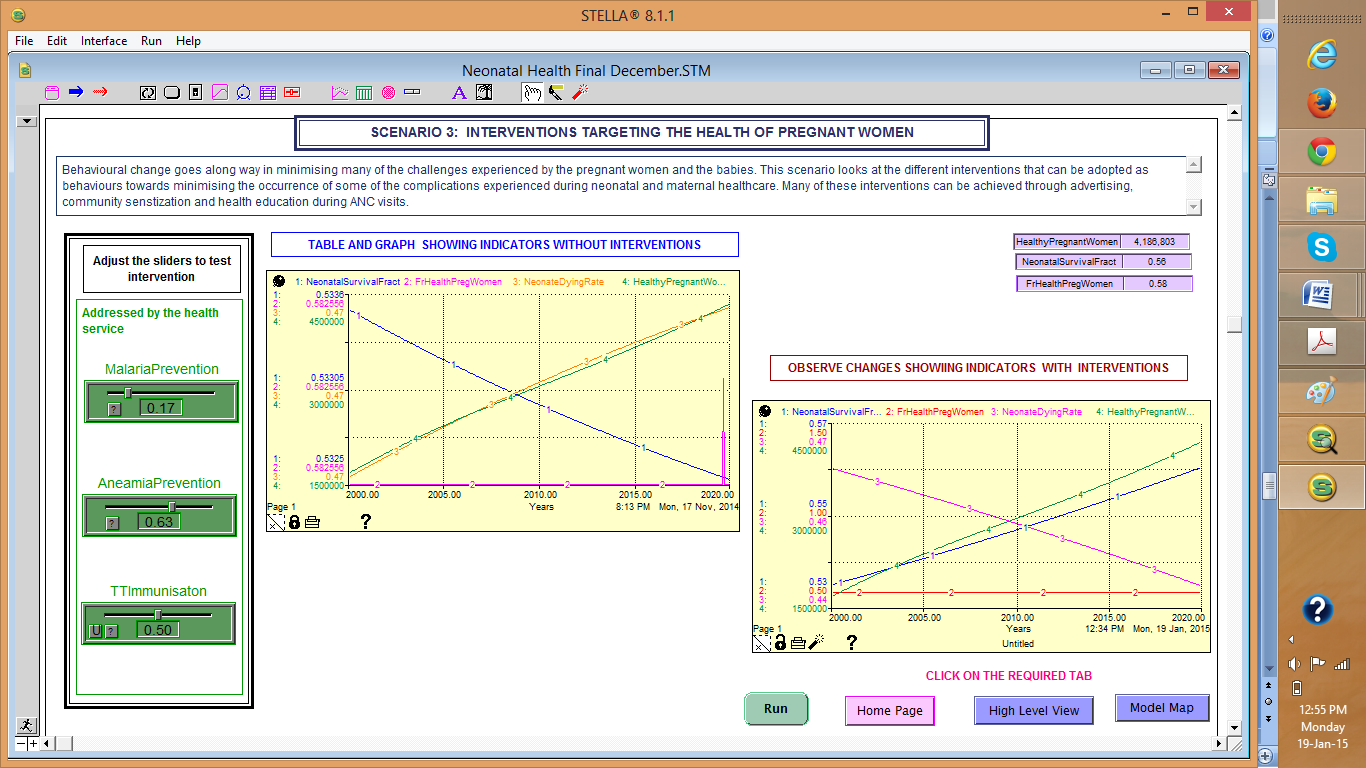


| **Instructions** |
| --- |
| Use sliders to adjust the input variables, Run the model.  |
| **Provide comments about the behavior and dynamics displayed in the tables and graphs.** |
|  |

1. **Validity** *(please tick)*
2. How do you rate this model as far as representation of the issues in neonatal healthcare service

[a]Very good [b]Good [c]Fairly good [d]Not at all good

1. Does the model appear to be a reasonable (realistic) imitation of a real world system?

[a]Very reasonable [b]Reasonable [c]Fairly reasonable [d]Not reasonable

1. Does the model capture and communicate issues in neonatal healthcare service

[a]Very good [b]Good [c]Fairly good [d]Not at all good

1. Do you think this model is a useful communication tool concerning neonatal healthcare issues [a]Very useful [b]Useful [c]Fairly useful [d]Not at all
2. Do you think this model is a tool that can be used by stakeholders in decision making

[a]Very useful [b]Useful [c]Fairly useful [d]Not at all

1. Provide comments on the model (strengths, weaknesses) as well as suggestions that could improve the model.

|  |
| --- |

*Thank you*
